# Acute heat stress upregulates *Akr1b3* through *Nrf-2* to increase endogenous fructose leading to kidney injury

**DOI:** 10.1016/j.jbc.2024.108121

**Published:** 2024-12-21

**Authors:** Shuai Wang, Xuan Pang, Yujuan Cai, Xue Tian, Jingyi Bai, Mingchuan Xi, Jiaxue Cao, Long Jin, Xun Wang, Tao Wang, Diyan Li, Mingzhou Li, Xiaolan Fan

**Affiliations:** 1 State Key Laboratory of Swine and Poultry Breeding Industry, Sichuan Agricultural University, Chengdu, China; 2 Livestock and Poultry Multi-omics Key Laboratory of Ministry of Agriculture and Rural Affairs, College of Animal Science and Technology, Sichuan Agricultural University, Chengdu, China; 3 Farm Animal Genetic Resources Exploration and Innovation Key Laboratory of Sichuan Province, Sichuan Agricultural University, Chengdu, Sichuan, China; 4 Antibiotics Research and Re-evaluation Key Laboratory of Sichuan Province, Sichuan Industrial Institute of Antibiotics, School of Pharmacy, Chengdu University, Chengdu, China

**Keywords:** heat stress, *Nrf-2*, *Akr1b3*, fructose, kidney

## Abstract

In recent years, the prevalence of extremely high-temperature climates has led to an increase in cases of acute heat stress (HS), which has been identified as a contributing factor to various kidney diseases. Fructose, the end product of the polyol pathway, has been linked to kidney conditions such as kidney stones, chronic kidney disease, and acute kidney injury. However, the relationship between acute HS and kidney injury caused by endogenous fructose remains unclear. The study found that acute HS triggers the production of reactive oxygen species, which in turn activate the *Nrf-2* and *Akr1b3* leading to an increase in endogenous fructose levels in kidney cells. It was further demonstrated that the elevated levels of endogenous fructose play a crucial role in causing damage to kidney cells. Moreover, inhibiting *Nrf-2* effectively mitigated kidney damage induced by acute HS by reducing endogenous fructose levels. These findings underscore the detrimental impact of excessive fructose resulting from acute stress on kidney function, offering a novel perspective for future research on the prevention and treatment of acute HS–induced kidney injury.

Global warming is accelerating, and according to existing reports, the average maximum temperature has increased by 0.8 °C from 1969 to 2019 (1). With the temperature rising, the frequency of extremely high temperatures increased markedly. Moreover, the number of individuals affected by extreme heat conditions has risen considerably since 2010 (2, 3, 4). It is worth noting that studies have predicted that the global temperature will continue to rise in the next few decades, which will significantly increase the morbidity and mortality associated with acute heat stress (HS) (1, 5, 6, 7).

The kidney plays a crucial role in protecting the stability of the human internal environment (1, 8). When subjected to HS, the kidney will protect the body from HS damage and maintain the stability of osmotic pressure, enhancing reabsorption to avoid dehydration and heat stroke. Exposure to high temperatures beyond the kidney's limits can result in various kidney-related diseases, and acute kidney injury due to acute HS is a significant factor leading to kidney failure (9, 10, 11, 12, 13, 14). Repeated exposure to HS and dehydration also raises the risk of chronic kidney disease (15, 16, 17, 18); HS is a common cause of nephrolithiasis (19, 20, 21). Moreover, acute HS can harm the glomerulus and kidney tubules, directly, potentially causing inflammation (22).

Fructose is a ubiquitous monosaccharide in the biological realm, serving as a crucial component of both metabolic processes and growth. Additionally, it represents a significant constituent of modern human diets, ranking second only to glucose in terms of blood sugar content (23, 24). Excessive fructose intake has several negative effects on human physiology, making humans susceptible to cardiometabolic disease, insulin resistance, obesity, and nephrolithiasis (21). However, in addition to the fructose that enters the body through food, the production of fructose in the kidney is a very important causative factor in kidney disease (25). The production of endogenous fructose is mainly through the polyol pathway, which consists of two enzymes, aldose reductase (AR) and sorbitol dehydrogenase (SORD) (26). In this pathway, glucose is used as the starting material, and AR produces sorbitol as an intermediate. Next, SORD produces fructose, the end product of the polyol pathway (27, 28). AR, a member of the aldo-keto reductase family, is the key rate-limiting enzyme in the polyol pathway and plays a crucial role in the production of endogenous fructose (29).

NRF-2 is an important nuclear transcription factor in response to oxidative stress in cells. When cells are stimulated, NRF-2 enters the nucleus to regulate the transcription of many genes and resist the influence of external stimuli (30, 31). In previous studies, it was found that intracellular reactive oxygen species (ROS) significantly increased and triggered oxidative stress after acute HS and the elevation of ROS activates NRF-2 (31, 32, 33). Meanwhile, NRF-2 is a crucial transcription factor in responding to oxidative stress triggered by excessive ROS (34). Experimental findings demonstrate that NRF-2, a key nuclear transcription factor responsive to stress, typically forms a heterodimer with sMaf protein and translocates into the nucleus to bind to antioxidant response elements (AREs), thereby modulating the transcriptional expression of downstream target genes of *Nrf-2* (35). Moreover, it should be noted that functional ARE was found in the upstream promoter region of the mouse AR gene (*Akr1b3*), and activated NRF-2 will enter the nucleus to recognize ARE and upregulate *Akr1b3* transcription (35, 36). Additionally, NRF-2 interacts with NFAT5 (nuclear factor of activated T cells 5) and AP-1 (a dimer composed of c-Jun and c-Fos) to form multiple stress response region acting as an enhancer to regulate AR expression o in mice (35, 37).

Both dietary fructose intake and endogenous fructose have been confirmed to be closely related to kidney disease. However, the relationship between endogenous fructose and kidney disease and the mechanism of fructose production after acute HS is not clear. Here, by in *vivo* in mice and cell experiments, we have elucidated the processes by which acute HS triggers the NRF2-AKR1B3 pathway, resulting in the upregulation of endogenous fructose and subsequent kidney injury. These findings offer valuable insights into the renal effects of acute HS.

## Result

### Acute HS increases ROS in mice's kidney

**Figure 1:**
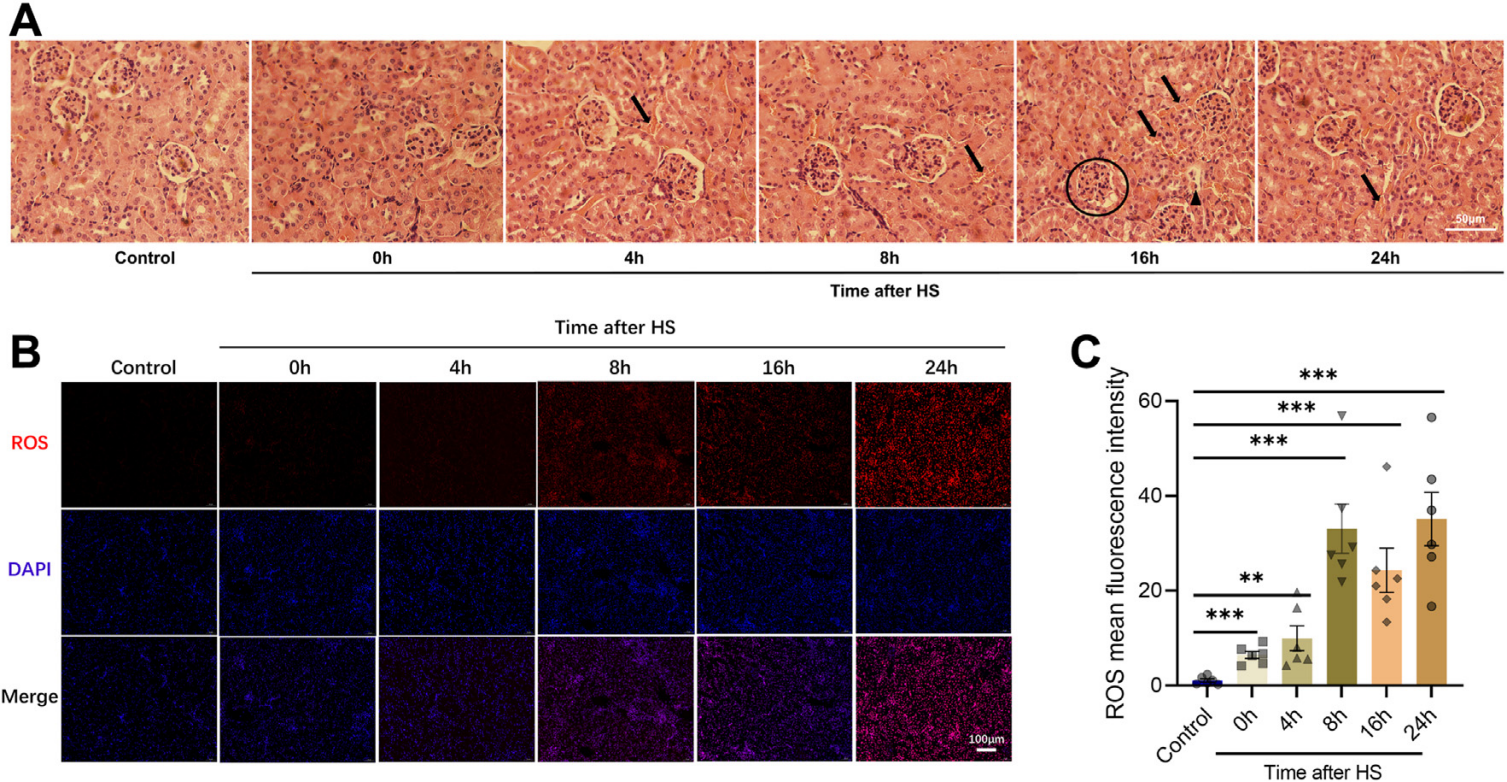
**Acute heat stress induces kidney injury and increases ROS.***A*, mice kidneys were stained with hematoxylin-eosin at different time points after HS (“↑”: hemorrhage in tissue; “Δ”: renal tubular injury; “O”: glomerular edema, and there were six mice in each group). *B–C*, ROS fluorescence intensity at different time points after HS (Error bars indicate SD, ∗∗*p* < 0.01, ∗∗∗*p* < 0.001; there were six mice in each group, and the images were selected from the same position on the kidney). HS, heat stress; ROS, reactive oxygen species.

To investigate the impact of acute HS on the kidney, we maintained mice in an environment with the air temperature at 42 °C and humidity at 40% ± 5 for 2 h to establish acute HS models and rectal temperature was significantly elevated (shown in Fig. S1*B*). After HS, we collected mice kidneys at 0h, 4h, 8h, 16h, and 24h (Fig. S1*A*). Kidney injuries were counted in kidney sections stained with hematoxylin and eosin (H&E) before and after acute HS treatment at 0h, 4h, 8h, 16h, and 24h (Fig. S1*C*). Pathological histology data reveal varying degrees of kidney injury in mice at various time points after exposure to HS. Specifically, 16 h post-HS, kidney sections stained with H&E showed significant damage (Fig. 1*A* and Fig. S1*D*). Previous studies have reported that HS increases cell ROS (33, 38, 39, 40), prompting us to measure fluorescence intensity in mouse kidneys. Our findings indicate a significant in ROS levels post-HS (Fig. 1, *B* and *C* and Fig. S1*E*), suggesting that acute HS leads to kidney injury and elevated ROS levels.

### Acute HS upregulates AR and increases endogenous fructose in mice kidney

**Figure 2:**
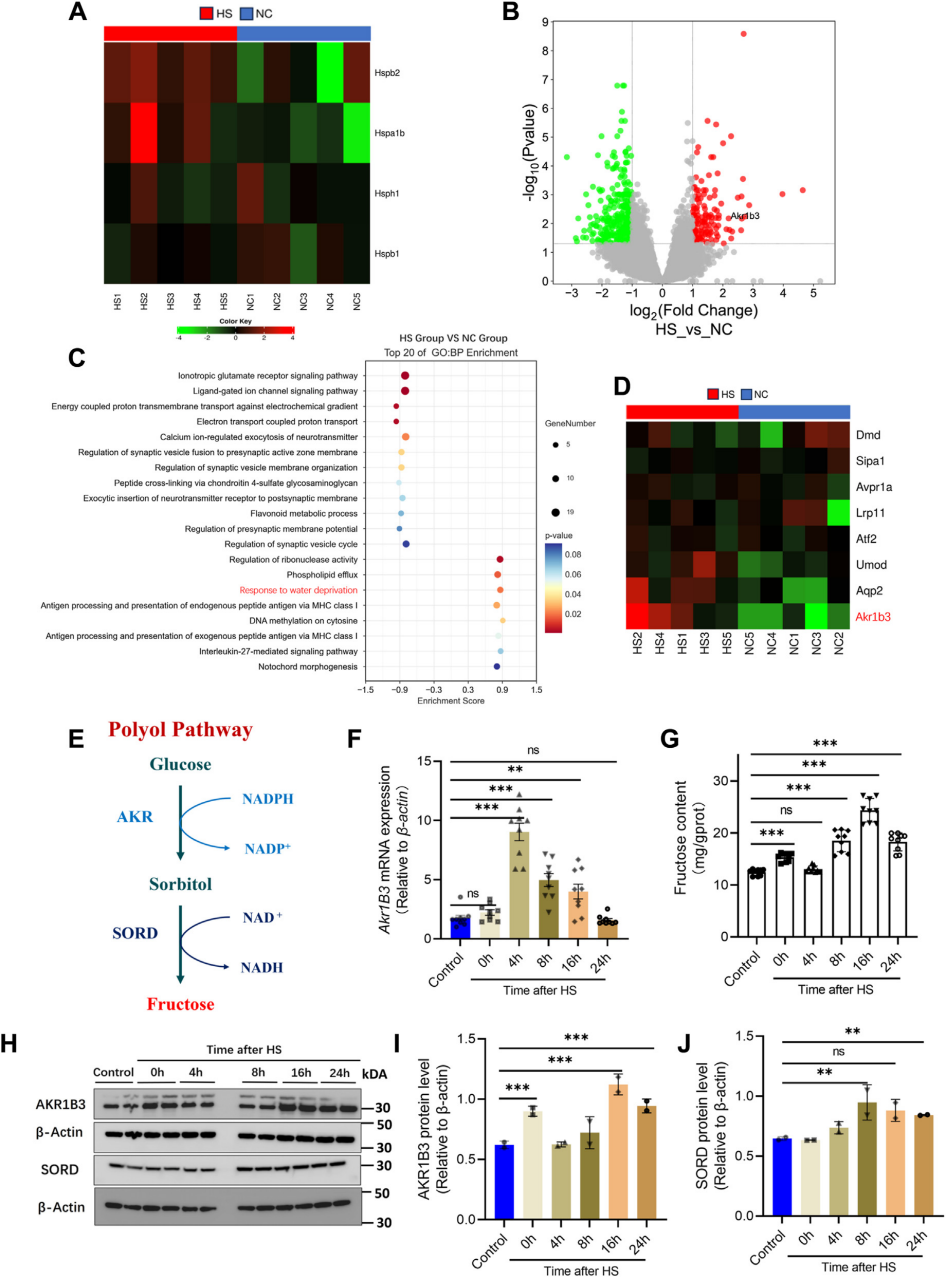
**Transcriptome data analysis.***A*, a heat map of heat stress–related protein expression changes. *B*, the number of differentially expressed genes in the HS group compared with the control group, 134 genes downexpression and 58 genes upexpression (*p* value<0.05, |log_2_fold change|≥1, there were five mice in each group). *C*, the top 20 enrichment of GO: BP pathway analysis between the control group and HS group. *D*, heat map of expression changes of enriched genes in response to water deprivation. *E*, schematic representation of the polyol pathway. *F*, *Akr1b3* mRNA expression of different time points post-HS (error bars indicate SD, ∗∗*p* < 0.01, ∗∗∗*p* < 0.001, and there were three mice in each group). *G*, fructose content of different time points after HS (error bars indicate SD, ∗∗∗*p* < 0.001, and there were three mice in each group). *H–J*, the protein expression levels of AKR1B3 and SORD were analyzed by Western blotting (error bars indicate SD, ∗*p* < 0.05, ∗∗*p* < 0.01). *Akr1b3, aldo-keto reductase family 1 member B3;* HS, heat stress; SORD, sorbitol dehydrogenase.

To investigate alteration at the transcription level in kidney following acute HS, a control group and a group assessed 16 h after HS were selected for RNA-seq analysis. The total raw read counts for the RNA-seq analysis are presented (Fig. S2), after filtering, a total of 15,196 genes were included in subsequent analysis. Moreover, we found that *Hspb2* (*heat shock protein 2*) and *Hspa1b* (*heat shock 70* kDa *protein 1 b*), which are associated with HS (41, 42, 43, 44, 45), were significantly increased (Fig. 2*A*). These results further verified the successful construction of the mouse HS model. Compared with the control group, a total of 192 differentially expressed genes were detected in the kidney tissue of the HS group, of which 134 genes were downregulated and 58 genes were upregulated (Fig. 2*B*, shown in supplementary data 1). Gene set enrichment analysis showed that the HS-responsive transcripts converged on pathways related to the response to the water deprivation process (Fig. 2*C*). Among the genes enriched in response to water deprivation process, we found the expression of *aldo-keto reductase family 1 member B3* (*Akr1b3*), the key rate-limiting enzyme in the polyol pathway, was significantly upregulated (25, 29) (Fig. 2*D*).

To confirm the results, the mRNA expression of mice kidneys was analyzed at various time points (0h, 4h, 8h, 16h, and 24h) post-HS. A significant increase in *Akr1b3* expression was observed after HS (Fig. 2*F*). *Akr1b3* is known to be the rate-limiting enzyme in the polyol pathway which is associated with glucose metabolism and fructose synthesis (25, 29). Additionally, SORD is another key enzyme in this pathway, as depicted in (Fig. 2*E*). Furthermore, the levels of fructose, the final product of the polyol pathway, were measured in mouse kidneys post HS. The results indicated an increase in kidney fructose content following heat exposure, peaking at 16 h (Fig. 2*G*). Subsequently, we examined the protein expression levels of both AKR1B3 and SORD in the polyol pathway within mouse kidneys. Our findings revealed that the protein level of AKR1B3 was significantly increased at 16h post-HS, whereas SORD showed only slight upregulation following heat exposure (Fig. 2, *H*–*J*). These findings suggest that acute HS stimulates endogenous fructose production in kidneys of mice by activating the polyol pathway.

Previous studies have shown that high levels of fructose in the kidneys can lead to glomerular hypertension and tubule interstitial damage (25, 46, 47, 48, 49). To more deeply understand the regulation of AKR1B3 expression by HS, we employed the kidney tubular epithelium cells (TCMK-1) and established an acute HS model (Fig. S3*A*). Cells were maintained at 42 °C and 5% CO_2_ environment for a different time, and the results revealed a significant increase in ROS fluorescence intensity over time, peaking at 2.5 h after HS treatment (Fig. S3, *B* and *C*). Consistent with the results of in *vivo* experiments in mice, acute HS also increased the protein expression of AKR1B3 and SORD and significantly increased endogenous fructose levels in TCMK-1 cells (Fig. S4, *A–D*).

Next, we wanted to explore whether the increase of fructose would affect cells. We calculated from the pre-experiment that the fructose elevation due to HS was approximately 2.5 μM. Then, we added the same amount of fructose to the cell culture medium and detected the change in TCMK-1 cell's vitality. Initially, TUNEL fluorescence staining was employed to detect the apoptosis of TCMK-1 cells, showing an increase in apoptosis levels after exposure to fructose medium for 24 and 48 h (Fig. S5, *A* and *B*). Subsequently, the viability of TCMK-1 cells was assessed using the Cell Counting Kit-8 assay, which indicated a significant decrease in cell viability following fructose treatment for 24 and 48 h (Fig. S5*C*). To further verify the effects of fructose on TCMK-1 cells, we chose to treat cells with different levels of fructose for 48 h and then measured cell viability. The results show that the cell viability decreased significantly with fructose concentration greater than 2.5 μM (Fig. S5*D*). This strongly demonstrates that endogenous fructose elevated by acute HS is sufficient to cause renal cell damage.

### Acute HS upregulates Nrf-2 expression

**Figure 3:**
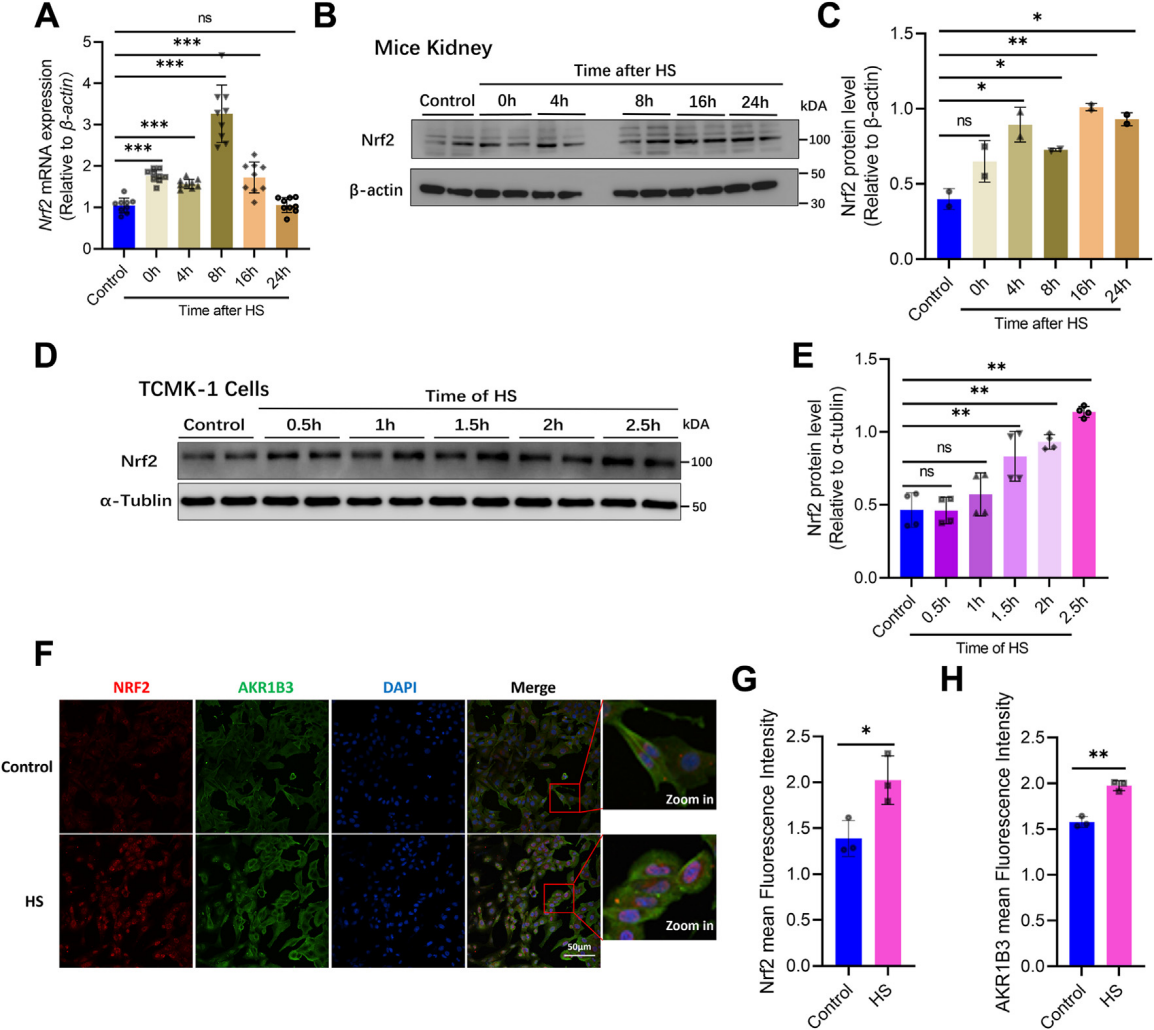
**Acute heat stress upregulates *Nrf-2* expression.***A*, *Nrf-2* mRNA expression change in the kidney between the control group and 0h, 4h, 8h, 16h, and 24h after acute heat stress (error bars indicate SD, ∗∗∗*p* < 0.001). *B–C*, NRF-2 protein expression change in the kidney between the control group and 0h, 4h, 8h, 16h, and 24h after acute heat stress (the β-actin gel blots reused with the fourth row of Figure 2*H* for the same analysis, and error bars indicate SD, ∗*p* < 0.05, ∗∗*p* < 0.01). *D–E,* NRF-2 protein expression change in TCMK-1 cells between the control group and heat stress for 0.5 h, 1h, 1.5 h, 2h, and 2.5 h (error bars indicate SD, ∗∗*p* < 0.01). *F–G,* immunofluorescence of NRF-2 and AKR1B3 in the control group and HS group (acute heat stress 2.5 h) (error bars indicate SD, ∗*p* < 0.05, ∗∗*p* < 0.01, and mean fluorescence intensity related to the amounts of cells). HS, heat stress.

Transcriptional regulation mediated by the transcription factor *Nrf-2* represents a crucial response pathway in the context of ROS (50). Prior studies have indicated that *Akr1b3* may be a target gene of *Nrf-2* (35, 51); consequently, we focused on the expression of *Nrf-2* in cases of HS-induced kidney injury. The results revealed a significant increase in *Nrf-2* mRNA levels post-acute HS, peaking at 8 h posttreatment and returning to baseline levels by 24 h (Fig. 3*A*). Furthermore, the investigation of NRF-2 protein levels in the kidney following acute HS treatment showed an upward trend, with the highest level observed at 16 h posttreatment (Fig. 3, *B* and *C*).

To further investigate the impact of acute HS treatment on *Nrf-2* expression in cells, the TCMK-1 cell model was employed in this in *vitro* experiment. The results revealed a progressive increase in NRF-2 protein expression with the duration of acute HS treatment peaking at 2.5 h (Fig. 3, *D* and *E*). Subsequently, quantification of NRF-2 and AKR1B3 fluorescence intensity in TCMK-1 cells using immunofluorescence showed a significant enhancement in both proteins in the group subjected to acute HS for 2.5 h (Fig. 3, *F–H*). Additionally, a notable increase in NRF-2 accumulation in the nucleus was observed postacute HS treatment (Fig. 3*F*).

It has been proved in the above experiments that acute HS can stimulate a large number of ROS production in *vivo* and in *vitro*, leading to the upregulation of *Nrf-2* (33, 38, 39, 40, 52). To further investigate the role of ROS in activating *Nrf-2* to regulate *Akr1b3* and enhance the endogenous production of fructose in TCMK-1 cells, we used menadione, a polycyclic aromatic ketone compound that serves as a precursor for vitamin K synthesis and generates intracellular ROS at several cellular sites through redox cycling (53, 54, 55, 56). A concentration gradient of menadione ranging from 0 μM to 20 μM was established, with 5 μM determined as the optimal concentration based on ROS fluorescence intensity (Fig. S6, *A* and *B*). Subsequent analysis revealed that ROS notably increased the intracellular expression of *Nrf-2* and *Akr1b3* (Fig. S6, *C* and *D*). Moreover, the intracellular fructose content was significantly elevated in TCMK-1 cells treated with 5μM menadione compared to the control group (Fig. S6*E*).

In summary, acute HS elevated the ROS content in kidney and TCMK-1 cells, leading to the upregulation of *Nrf-2* and *Akr1b3* expression and enhancing intracellular fructose content.

### Nrf-2 plays a pivotal role in regulating of kidney endogenous fructose production during acute HS

**Figure 4:**
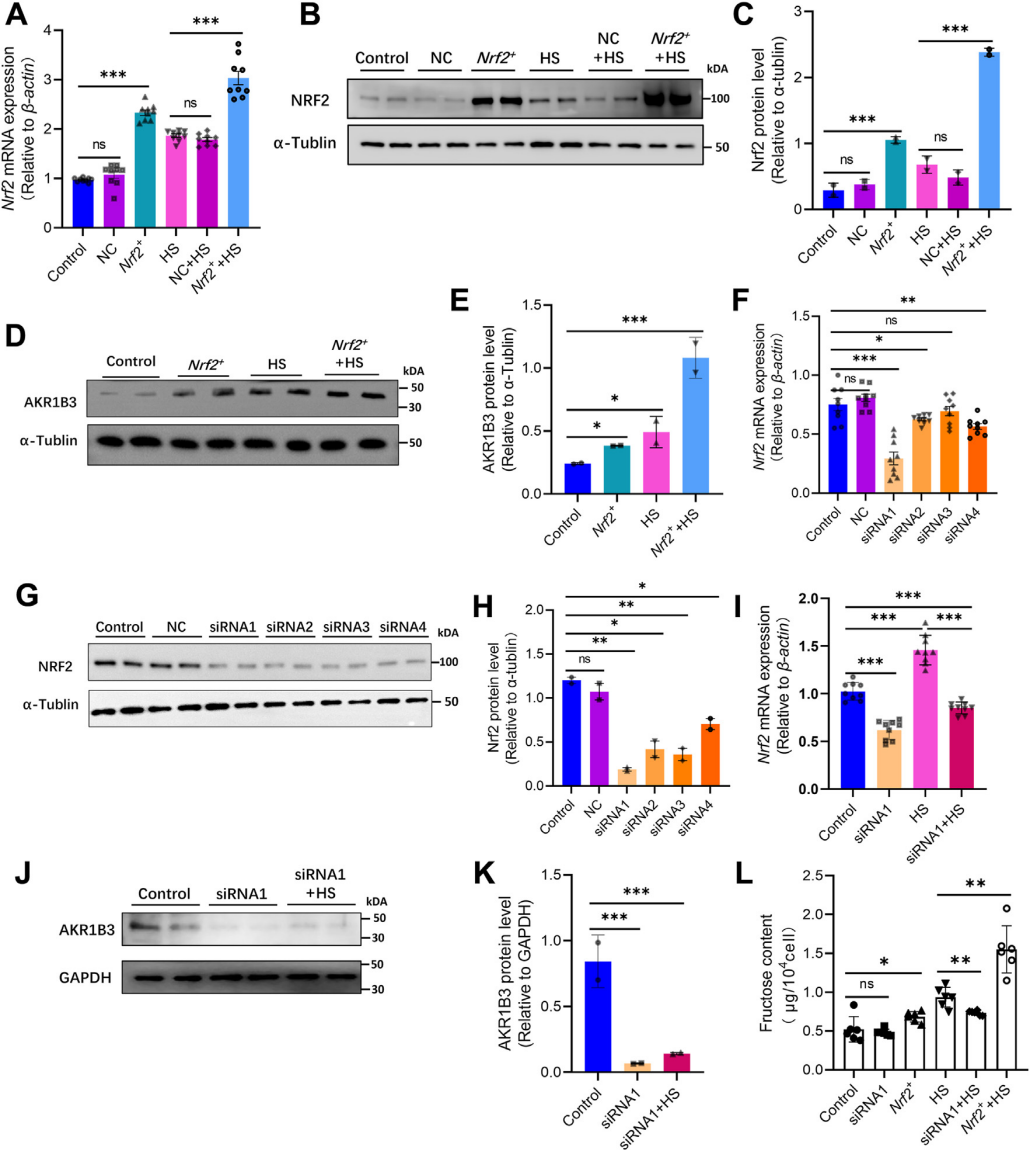
***Nrf-2* is a key factor in the regulation of renal endogenous fructose production in response to acute heat stress.***A*, after *Nrf-2* overexpression the mRNA expression changes in TCMK-1 cells between different groups (error bars indicate SD, ∗∗∗*p* < 0.001). *B–C*, after *Nrf-2* overexpression the protein expression change in TCMK-1 cells between different groups (error bars indicate SD, ∗*p* < 0.05, ∗∗∗*p* < 0.001). *D–E*, after *Nrf-2* overexpression, the AKR1B3 protein expression changes in TCMK-1 cells between different groups (error bars indicate SD, ∗*p* < 0.05, ∗∗*p* < 0.01). *F*, after four siRNA sequences transfected into TCMK-1 cells, the *Nrf-2* mRNA expression change (error bars indicate SD, ∗∗∗*p* < 0.001). *J–K*, AKR1B3 protein expression change between the control, siRNA1, and siRNA1+HS group (error bars indicate SD, ∗*p* < 0.05, ∗∗*p* < 0.01). *L*, the fructose content of TCMK-1 cells in different groups (error bars indicate SD, ∗*p* < 0.05, ∗∗*p* < 0.01). AKR1B3, aldo-keto reductase family 1 member B3.

To investigate the regulatory relationship between *Nrf-2* and *Akr1b3*, an *Nrf-2* overexpression model was established in TCMK-1 cells. The *Nrf-2* mRNA and protein expression significantly increased in the *Nrf-2* overexpression group compared to the negative control group (Fig. 4, *A*–*C*). Moreover, the NRF-2 protein expression was notably higher in the HS group than in the normal temperature group (Fig. 4, *B* and *C*). Notably, the *Nrf-2* overexpression combined with the HS group showed exceptionally elevated levels of AKR1B3 compared to other groups (Fig. 4, *D* and *E*). These results suggest that *Nrf-2* plays a crucial role in regulating AKR1B3 during acute HS. To further confirm the regulatory relationship between *Nrf-2* and *Akr1b3*, a *Nrf-2* knockdown model was constructed in TCMK-1 cells. Using the *Nrf-2* sequence (NM_010902.5) as a template, four siRNA sequences were generated and transfected into TCMK-1 cells. The knockdown efficiency was assessed by measuring the mRNA and protein levels of NRF-2. Results showed that siRNA1 exhibited the highest knockdown efficiency, making it suitable for subsequent experiments (Fig. 4, *F–H*). Subsequent analysis revealed a significant decrease in *Nrf-2* mRNA expression in TCMK-1 cells that were subjected to acute HS, in the siRNA1+HS (with both *Nrf-2* knockdown and HS) group compared to the HS group (Fig. 4*I*). Subsequently, the protein levels of AKR1B3 were examined in the control group, siRNA1 group, and siRNA1+HS group, showing a significant decrease in AKR1B3 expression after *Nrf-2* knockdown (Fig. 4, *J* and *K*).

The intracellular fructose content was measured in various experimental groups, and results showed that the overexpression of *Nrf-2* significantly increased the fructose level compared to the control group, with further elevation observed after acute HS. There was no significant difference in fructose levels between the control group and the siRNA1 group. However, the fructose levels in the siRNA1+HS group were significantly lower than those in the HS group (Fig. 4*L*). These findings suggest that *Nrf-2* plays a crucial role in both upregulating AKR1B3 under acute HS conditions and increasing endogenous fructose production in the kidney.

### Inhibiting the Nrf-2 will recover the kidney damage caused by acute HS

**Figure 5:**
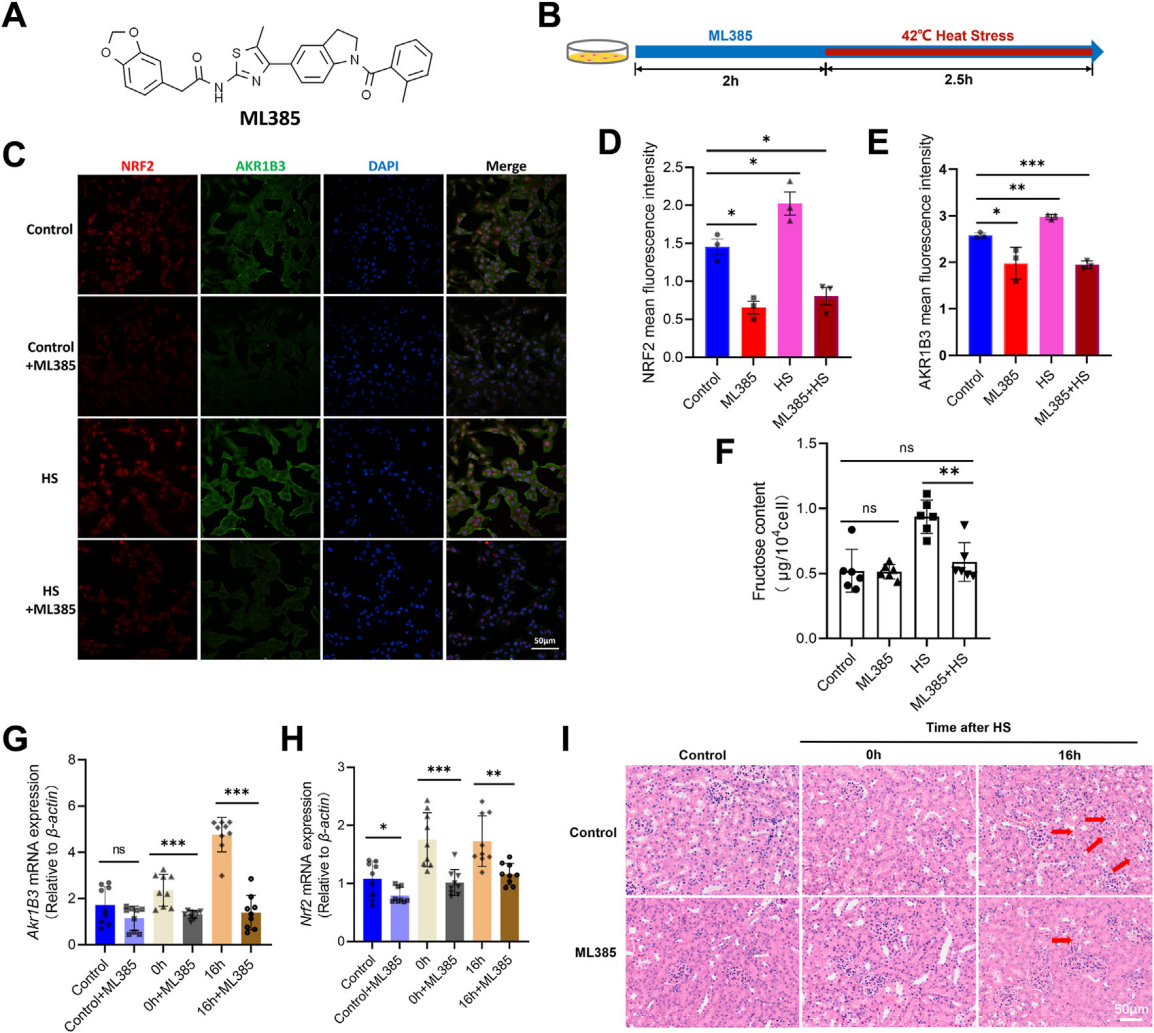
**Inhibition of *Nrf-2* restores endogenous fructose production induced by acute heat stress and cell viability after fructose treatment.***A*, molecular structure of ML385. *B*, flow chart of experimental treatment of TCMK-1 cells supplemented with ML385. *C–E*, immunofluorescence detection of NRF-2 and AKR1B3 in different treatment groups (error bars indicate SD, ∗*p* < 0.05, ∗∗*p* < 0.01, ∗∗∗*p* < 0.001, and mean fluorescence intensity related to the amounts of cells). *F*, changes of fructose content in TCMK-1 cells in different treatment groups (error bars indicate SD, ∗∗*p* < 0.01, and there were six mice in each group). *G–H, Nrf-2* and *Akr1b3* mRNA levels were tested in different treatment groups (error bars indicate SD, ∗*p* < 0.05, ∗∗*p* < 0.01, ∗∗∗*p* < 0.001, and there were three mice in each group). *I*, kidney injury of mice in different treatment groups,“↑”: hemorrhage in tissue (there were three mice in each group). *Akr1b3, aldo-keto reductase family 1 member B3.*

To further validate our experimental findings, we used ML385 (Fig. 5*A*), a proven inhibitor of *Nrf-2* (57). Based on the research results of published papers (57, 58, 59), 5μM ML385 was selected as the concentration for subsequent experiments. TCMK-1 cells were pretreated with a complete medium containing 5μM ML385 at a concentration of for 2 hours before exposure to acute HS. After this pretreatment, the cells were placed in subjected to a heat-stress environment for 2.5 h, as depicted in Fig. 5*B*. Subsequently, the cells were then categorized into four groups based on the medium components and treatment conditions: Control, Control + ML385, HS, and HS + ML385. Immunofluorescence analysis revealed a significant decrease in AKR1B3 expression after the *Nrf-2* inhibition (Fig. 5, *C–E*). A notable recovery in fructose content was observed in the HS + ML385 group compared to the acute HS group (Fig. 5*F*). Moreover, the fructose content in the HS + ML385 group did not significantly differ from the control group. These experiments highlight that inhibiting *Nrf-2* can normalize the elevated intracellular fructose levels induced by acute HS.

To explore whether *Nrf-2* inhibition could restore acute HS-induced kidney injury, ML385 was injected into wild-type *C57BL/6J* mice. In this study, we conducted a pre-experimental validation based on the findings of previously published papers (60, 61). ML385 (30mg/kg) was used for intraperitoneal injection in the time 45 to 60 min before acute HS treatment (Fig. S7*A*). Kidney samples of mice before acute HS and at 0h and 16h after acute HS treatment were collected as material for subsequent experiments. Meanwhile, a vehicle without ML385 was used as a negative control (6% DMSO+40% PEG300 + 5% Tween 80 + 49% ddH_2_O). In this study, we next tested the mRNA expression of *Nrf-2* and *Akr1b3* at different time points with different treatments, both of the two genes were significantly decreased when treated by ML385 in mice kidneys (Fig. 5, *G* and *H*). At 16 h after HS, H&E staining showed that ML385 injection reduced interstitial damage in kidney tissue, but tissue swelling remained (Fig. 5*I* and Fig. S7*B*). Next, kidney injuries were counted in kidney sections stained with hematoxylin and eosin of different treatments (Fig. S7*C*). This suggests that inhibition of *Nrf-2* can alleviate kidney injury in mice after HS to a certain extent.

## Discussion

This study utilized mice and TCMK-1 cell models to explore the central mechanism behind acute HS-induced kidney injury. The findings suggest that acute HS can enhance *Nrf-2* expression, leading to the regulation of the downstream target gene *Akr1b3* and subsequently influencing endogenous fructose production. The results highlight the dual role of *Nrf-2* as a crucial transcription factor in both AKR1B3 expression and the modulation of fructose production under acute HS conditions.

### Endogenous fructose and kidney injury

Fructose intake increases the risk of kidney-related diseases (62, 63), and the metabolism of fructose can generate uric acid which also causes many diseases in kidney (64, 65). In previous studies, the intake of 150 g of fructose per day (equivalent to four and a half 20-ounce bottles of cola) has been identified as a potentially excessive amount for humans, particularly when consumed over an extended period, and this level of intake has been linked to the development of severe diseases (21, 66, 67). Interestingly, the comparison of the endogenous fructose with the intake indicates that the endogenous fructose is relatively insignificant, but endogenous fructose also plays a very important role in kidney-related diseases (68, 69). Moreover, the existing literature has concentrated on the effects of external fructose intake, with a paucity of studies examining the impact of endogenous fructose on kidney damage, and the related mechanism is even less.

The polyol pathway is activated in acute kidney injury, leading to increased endogenous fructose production, exacerbating kidney injury and triggering oxidative stress in the kidney (68). In chronic kidney disease, fructose can elevate protein levels in urine by impacting the glomerular filtration rate, and in long-term chronic kidney disease, it may result in complications such as anemia due to reduced erythropoietin production by the kidneys (70, 71), iron deficiency in the body due to reduced red blood cell viability (72, 73), and a mineral bone disease caused by disorders of vitamin D, calcium, and phosphate metabolism (74). Moreover, fructose can increase urinary stone formation through its effects on uric acid metabolism and urine pH (21). In addition to kidney stones, increased uric acid leads to overproduction and deposition of urate crystals in the urinary tract, leading to hyperuricemia (75). In our results, we found that after acute HS treatment, the polyol pathway is activated, and fructose is elevated both in *vivo* and in *vitro*, coinciding with kidney tissue damage. Moreover, we also demonstrated that elevated fructose in response to acute HS could reduce cell viability *in vitro*. These results suggest that elevated endogenous fructose is associated with kidney injury.

### Mechanisms of how acute HS regulates endogenous fructose in kidney

Previous studies have found that HS can increase intracellular ROS in many ways. First, HS can activate the inflammatory response and increase inflammatory cytokines, such as IL-1β, IL-18, and IL-6, which can further stimulate ROS production (76, 77). Second, HS also can cause an imbalance in the cellular REDOX state, resulting in reduced activity of antioxidant defense systems, such as superoxide dismutase, glutathione peroxidase, and catalase, which accumulate ROS in cells (78, 79). Mitochondrial ROS, as one of the main sources of cellular oxidative stress, is also affected by HS (32, 33). It has been reported that HS affects the electron transport chain of mitochondria (33) and induces mitochondrial membrane potential depolarization and mitochondrial dysfunction (80), which promote ROS generation in cells. In addition, HS also increases the expression of NRF-2/HO-1, the oxidative stress marker, which may be related to the cell response to the increased ROS (81). Furthermore, under typical physiological circumstances, the NRF2-KEAP1 protein is situated within the cytoplasm in an inactive configuration, directing NRF-2 toward degradation in order to maintain a relatively low NRF-2 activity state (82). However, under conditions of oxidative stress, NRF-2 and KEAP1 dissociate, and NRF-2 enters the nucleus to regulate its downstream genes, such as HO-1 (83, 84, 85). Interestingly, this study also found the above results, and after the TCMK-1 cells were exposed to HS, NRF-2 accumulated in the nucleus (Fig. 3*F*).

In this study, the intracellular ROS level was verified to increase in mice kidneys and TCMK-1 cells after acute HS. In addition, cellular immunofluorescence experiments showed that acute HS increased the nuclear accumulation of *Nrf-2*. In this study, cell models of *Nrf-2* overexpression and knockdown were constructed to confirm the role of *Nrf-2* in regulating AKR1B3. It was found that *Nrf-2* did not promote AKR1B3 expression in the absence of HS, but when *Nrf-2* expression was blocked, AKR1B3 was significantly inhibited, and endogenous fructose production was inhibited. After the addition of the *Nrf-2* inhibitor ML385, the expression of *Nrf-2* was decreased, nuclear aggregation of *Nrf-2* was significantly reduced, and the expression of AKR1B3 was inhibited. The acute heat-induced increase in endogenous fructose was also reversed by the addition of ML385.

Existing studies found that excessive fructose intake causes kidney damage by increasing ROS in the kidneys, and *Nrf-2* plays a protective role in this process (86, 87). Moreover, the metabolism of fructose also affects the nuclear translocation of *Nrf-2* (88). These results seem like the protective role of *Nrf-2* occurs under the condition of high fructose intake, but how *Nrf-2* affects endogenous fructose production is still lacking. In our study, acute HS can activate the function of *Nrf-2*, which upregulate the polyol pathway and endogenous fructose production. Moreover, the findings offer new insights into the effects of HS on the kidney.

**Figure 6:**
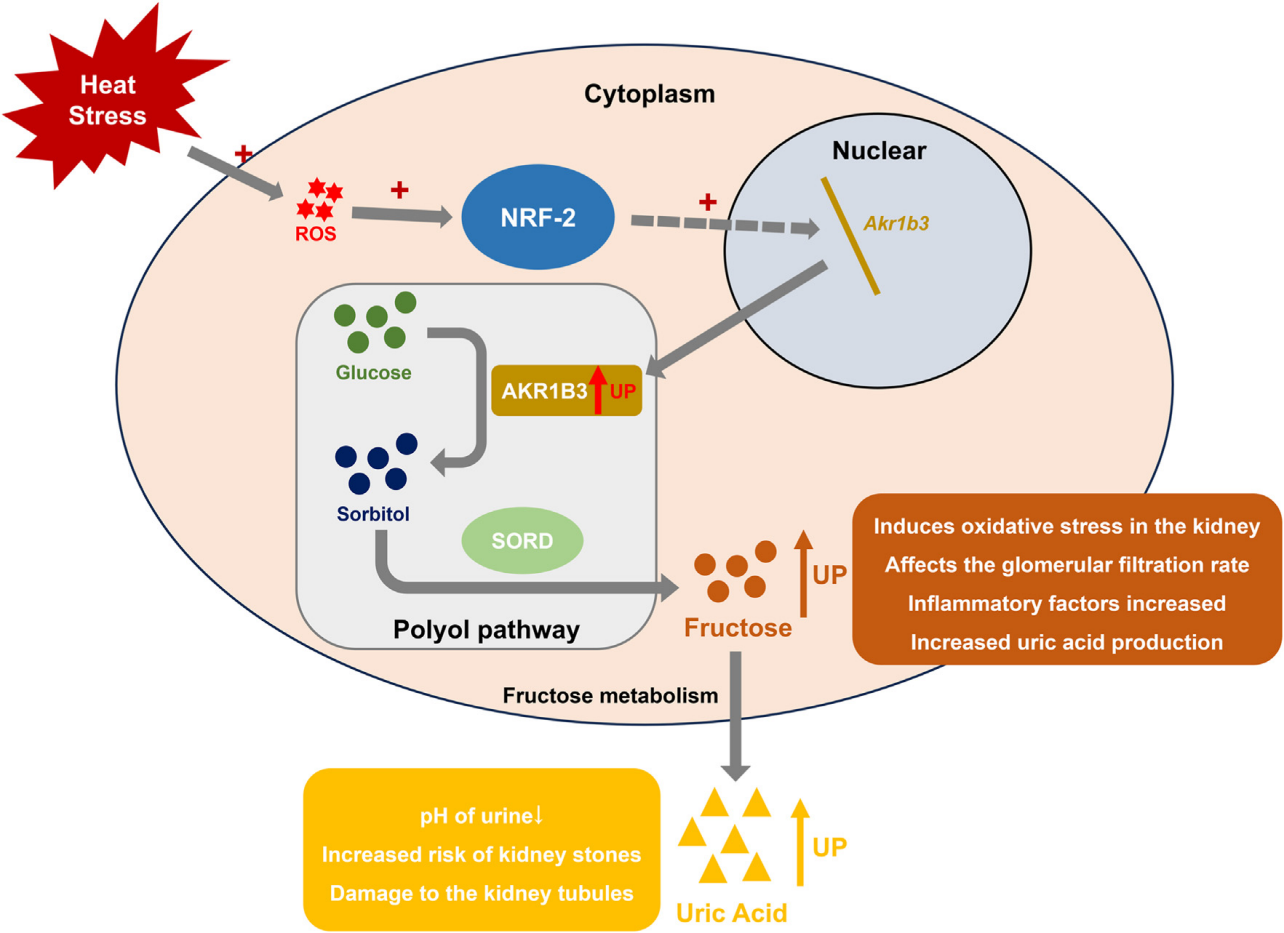
**Mechanisms of acute heat stress***–***regulated kidney endogenous fructose.** Acute heat stress can induce renal injury by generating endogenous fructose through the actions of NRF-2 and AKR1B3. ROS, reactive oxygen species; AKR1B3, aldo-keto reductase family 1 member B3.

In conclusion, as illustrated in Fig. 6, this study demonstrates that acute HS upregulates the expression of NRF-2, which subsequently translocates to the nucleus to activate the transcriptional expression of *Akr1b3*. The upregulation of AKR1B3 stimulates the intracellular polyol pathway, leading to an increase in endogenous fructose levels within renal cells. This mechanism may contribute to the observed kidney damage associated with acute HS. Our findings offer valuable insights for the prevention of acute HS-induced kidney injury at elevated temperatures.

## Experimental procedures

### Mice

Wildtype C57BL/6J female mice at 8 weeks were purchased from Dossy Experimental Animals Co., Ltd. Animals had free access to water and food. All experimental protocols involving animals were approved by the Institutional Animal Care and Use Committee.

### Cell culture

Mouse kidney tubular epithelium cells (TCMK-1) were purchased from Servicebio (STCC20015G). Cells were cultured in 95% Dulbecco's modified Eagle's medium (Sangon, E600003–0500), 5% FBS (Yeasen, 40131ES76), and 0.5% penicillin-streptomycin (Yeasen, 60162ES76). Cells were cultured at 37 °C in a humidified 5% CO_2_ chamber (Thermo Fisher Scientific).

### Antibodies and reagents

The primary antibodies used were as follows: Alpha tubulin (Proteintech, 66,031), GAPDH (Servicebio, GB11002), AKR1B3 (Bioss, bs-2405R), NRF2 (Cell Signaling Technology, 12721), SORD (Proteintech, 15,881), and DAPI (G1012, Servicebio). ML385 was purchased from TargetMol (846557–71–9). Fructose was purchased from Aladdin (F108335). Cy3 (GB21303) and FITC (GB22301) were purchased from Servicebio.

### Western blotting

The total protein was isolated from female mouse tissues or cells in a buffer containing 20 mM Tris, pH 7.5, 1% Triton X-100, 1 mM EDTA, 1 mM EGTA, and protease/phosphatase inhibitors. The lysate was centrifuged, and 20 to 50 mg of the supernatant was stored in Laemmli sample buffer (containing 62.5 mM Tris, 2% SDS, 25% glycerol, 0.01% bromophenol blue, and 5% β-mercaptoethanol). Proteins were incubated at 100 °C for 5 min and immunoblotted. Samples were separated by SDS-PAGE and transferred to polyvinylidene fluoride membranes. The membrane was blocked with 5% skim milk, 20 mM Tris, 500 mM sodium chloride, and 0.5% Tween-20 for 2 h and detected with primary antibody. We loaded the same treatment in two lanes. The samples in these two lanes were obtained from different replicate samples. In the case of mouse tissue, six mice were pooled into two samples. As for cells, six wells of cells were pooled into two samples.

### Immunofluorescence staining

The cells were plated 1 day in advance to ensure that the degree of cell confluence was at 60 to 80%. The precool PBS (G0002, Servicebio) was used to clean cells, and 4% paraformaldehyde was used to fix cells; the PBS was used to clean cells and then 0.5% Triton X-100 (purchased from Solarbio, F8100) was used to treat cells; the PBS was used to clean cells. Next, the primary antibodies were incubated overnight (NRF-2 1:200; AKR1B3 1:100), and then Cy3 (1:100) or FITC (1:100) were chosen to avoid light treatment for 40 min. DAPI (G1012, Servicebio) was then applied for 15 min, after which PBS was used to wash the samples three times, with each wash lasting 5 min. Observation was carried out with Nikon confocal microscope.

### Heat stress

For acute HS treatment, TCMK-1 cells were maintained at 42 °C and a 5% CO_2_ environment for 2.5 h. Mice were maintained at 42 °C for 2 h (humidity at 50% ± 5). In the time of HS, mice were fasted but had free access to water.

### ROS detection

For kidney tissue, the frozen sections were rewarmed at room temperature, and the moisture was controlled. The circles were drawn around the tissue with a histochemical pen (G6100, Servicebio), and self-fluorescence quencher (G1221, Servicebio) was added for 5 min and was rinsed with water for 10 min. The ROS dye solution, dihydroethidium (D7008, Sigma Aldrich), was added to the ring and incubated at 37 °C in a dark incubator for 30 min. The slide was placed in PBS (G0002, Servicebio) and washed by shaking on the decolorizing shaker for three times, 5 min each time. DAPI dye solution (G1012, Servicebio) was added and incubated at room temperature away from light for 10 min. The slide was placed in PBS and washed by shaking on the decolorizing table for three times, 5 min each time. Anti-fluorescence (G1401, Servicebio) quenching sealing tablets were subsequently applied. Observations were conducted using the Pannoramic MIDI (3DHISTECH).

The cells were plated 1 day in advance to ensure a confluence was of 50 to 70%. First, DCFH-DA (G1706, Servicebio) was diluted with Earle’s Balanced salt solution (G4213, Servicebio) into working liquid (1:1000). Then the cells were cleaned using the precool PBS, and 1 ml working liquid was transferred into 6-well plate which is maintained at 42°C and 5% CO_2_ environment for 45 min. Next, the cella were cleaned for 2 to 3 times using precool PBS. Finally, the cells were observed with Olympus fluorescent inverted microscope.

### ML385 treatment

Through the pre-experiment, we chose 5μM ML385 to treat TCMK-1 cells for mechanism study, and the mice were intraperitoneally injected with ML385 (purchased from Selleck Chemicals), 30mg/kg (dissolved in 6% DMSO+40% PEG300 + 5% Tween 80 + 49% ddH_2_O) 45 to 60 min before acute HS treatment.

### Fructose content detection

For fructose content detection of mice kidney, 0.1g kidney sample was added to 1ml distilled water for grinding to obtain crude extract and then centrifuged at room temperature for 12,000*g* for 10 min, and the supernatant was taken for measuring. For fructose content detection of TCMKM-1 cells, cells were collected into a centrifuge tube and counted. The cells were centrifuged at 800*g* for 10 min. After the supernatant was removed, the cells were blown and mixed with 1ml PBS for ultrasonic disruption (SCIENTZ-IID) and then centrifuged at room temperature for 12,000*g* for 10 min, and the supernatant was taken for measuring. Fructose content detection kit (purchased from Grace Biotechnology Company, G0530W) was used to measure the fructose content of kidney and cells.

### Fructose treatment

The cells were plated 1 day in advance to ensure that the degree of cell confluence was at 30 to 50%. The fructose was diluted in complete medium with the final concentration of 2.5μM. milliliter of complete medium with fructose was added into each well of 6-well plate, and the cells were kept at 42°C and 5% CO_2_ environment for 24h or 48h.

### Transfection

For *Nrf-2* overexpression, pcDNA3.1 plasmid was used as a vector to construct an overexpressed plasmid, with the *Nrf-2* (NCBI Reference Sequence: NM_010902.5) sequence inserted. For *Nrf-2* knockdown, the siRNA sequences shown in Table 1. The day before transfection, cells were seeded per 6-well plate in 2 ml of complete medium. The cells were incubated at 37 °C and 5% CO_2_. On the day of transfection, following the *TransIntroe* EL Transfection Reagent (TransGen Biotech, FT201), 4 μg of overexpressed plasmid (with 4μl EL transfection reagent) or 250 pmol siRNA (with 5μl EL transfection reagent) was used per well. Follow-up experiments were carried out 24 h later.

**Table 1:** siRNA sequence

Name	Sense (5′-3′)	Antisense (5′-3′)
siRNA1	CAGGAGAAUUCCUCCCAAUTT	AUUGGGAGGAAUUCUCCUGTT
siRNA2	CACGCUGAAAGUUCAGUCUTT	AGACUGAACUUUCAGCGUGTT
siRNA3	CAGAAAUGGACAGCAAUUATT	UAAUUGCUGUCCAUUUCUGTT
siRNA4	CUCGCAUUGAUCCGAGAUATT	UAUCUCGGAUCAAUGCGAGTT

### RNA extraction, cDNA synthesis, and quantitative real-time PCR

RNA extraction kit of cell samples was purchased from Accurate Biology (AG21023). Mice kidney samples were lysed with TRIzol (TransGen Biotech, ET111), and total RNA was extracted and separated using chloroform and isopropanol and purified with ethanol. cDNA synthesis was carried out using the First Strand cDNA Synthesis Kit from Yeasen (11141ES10). Quantitative PCR was performed using Hieff UNICON Universal Blue qPCR SYBR Green Master Mix (Yeasen, 11184ES08). The cycling conditions were as follows: 95 °C for 10 min; 95 °C for 15 s; 60 °C for 60 s, and cycled 40 times. Primers (shown in Table 2) were obtained from Sangon.

**Table 2:** Primer sequences

Gene	Primers
*Nrf2*	CCTCACCTCTGCTGCAAGTA TCAAATCCATGTCCTGCTGGG
*Akr1b3*	GTAACGTGCAGCGATCATGG AGTTGTTCCATAGCCGTCCA
*β-actin*	GCCTTCCTTCTTGGGTATGGAA CAGCTCAGTAACAGTCCGCC

### RNA-seq and differential gene expression analysis

RNA concentration was quantified using the Qubit (Thermo Fisher Scientific), and its quality was assessed using the Agilent 2100 Bioanalyzer (Agilent Technologies). A sample that passed RNA-seq data quality control and had a minimum RNA integrity number score of 7 was sent for sequencing. Library construction was performed using 2 μg RNA per sample. Briefly, mRNA was purified from total RNA using poly-T oligo-attached magnetic beads. Fragmentation was carried out using the fragmentation buffer. The first-strand cDNA was synthesized, and the second-strand cDNA synthesis was subsequently performed. The remaining overhangs were converted into blunt ends. After adenylation of the 3′ ends of DNA fragments, adaptors with a hairpin loop structure were ligated. Subsequently, PCR was performed. The resulting libraries were quality-controlled and sequenced on the DNBSEQ-G400 platform to generate 150 bp paired-end reads, according to the manufacturer’s instructions. Raw data were trimmed by Trim Galore (v.0.6.7) with default settings and then aligned to the mouse genome (GRCm39) with STAR (v.2.7.10a). The read count was generated by subread84 (v.2.0.1) with default parameters, and the transcripts per million of genes were quantified using RSEM (v.1.3.1). Differential expression genes analysis was performed in R using DESeq2. Genes with an |log_2_FoldChange|≥1 and a *p*-value < 0.05 were counted as differentially expressed genes. The R package, clusterProfiler, was used for the enrichment of differential expression genes. For Gene Ontology enrichment in terms of biological process, we used the simplify function of clusterProfiler to remove redundant enriched terms. Gene count of each mice in different groups was shown in Supplementary data 1. Differential expression analysis of transcriptomic data is elaborated in Supplementary data 2 for further details.

### Quantification and statistical analysis

All statistical analyses were carried out using GraphPad Prism 8.00 software. Measurements represent the mean, and error bars represent the standard deviation. As appropriate, Student’s unpaired 2-tailed *t* test or one-way ANOVA with *post hoc* Dunnett’s test was used to calculate significance. For all tests, *p* < 0.05 was considered significant. Asterisks reflecting the calculated *p* values are shown above each measurement, and ns indicates that differences between measurements were not statistically significant.

## Data availability

The RNA-seq dataset has been deposited in CNSA (https://db.cngb.org/cnsa/), under accession code CNP0005763 (mice kidney RNA-seq after acute heat stress).

## Supporting information

This article contains supporting information.

## Conflict of interest

The authors declare that they have no conflicts of interest with the contents of this article.

## Supporting information

Supporting information

Original blots
