# Supplementary material for: Acute heat stress upregulates Akr1b3 through Nrf-2 to increase endogenous fructose leading to kidney injury
Source: J Biol Chem. 2024 Dec 21;301(2):108121. doi: 10.1016/j.jbc.2024.108121 (PMC11834071; doi:10.1016/j.jbc.2024.108121)
Supplement: Supporting information [file mmc2.docx]

**Supporting Information**

Acute heat stress upregulates *Akr1b3* through *Nrf-2* to increase endogenous fructose leading to kidney injury

Shuai Wang^1#^, Xuan Pang^1#^, Yujuan Cai^1^, Xue Tian^1^, Jingyi Bai^1^, Mingchuan Xi^1^, Jiaxue Cao^1,2,3^, Long Jin^1,2,3^, Xun Wang^1,2,3^, Tao Wang^4^, Diyan Li^4^, Mingzhou Li^1,2,3*^, Xiaolan Fan^1,2,3*^

1. State Key Laboratory of Swine and Poultry Breeding Industry, Sichuan Agricultural University, Chengdu, 611130, China.

2. Livestock and Poultry Multi-omics Key Laboratory of Ministry of Agriculture and Rural Affairs, College of Animal Science and Technology, Sichuan Agricultural University, Chengdu, 611130, China

3. Farm Animal Genetic Resources Exploration and Innovation Key Laboratory of Sichuan Province, Sichuan Agricultural University, Chengdu, Sichuan, China

4. Antibiotics Research and Re‐evaluation Key Laboratory of Sichuan Province, Sichuan Industrial Institute of Antibiotics, School of Pharmacy, Chengdu University, Chengdu, China

# These authors have contributed equally to this work

*Correspondence

Prof. Mingzhou Li, Dr Xiaolan Fan

State Key Laboratory of Swine and Poultry Breeding Industry, Sichuan Agricultural University, Chengdu, 611130, China.

Tel: (86)28 86291010;

Fax: (86)28 86290987;

E-mail: mingzhou.li@sicau.edu.cn; xiaolanfan@sicau.edu.sn

**Supplementary Figures**


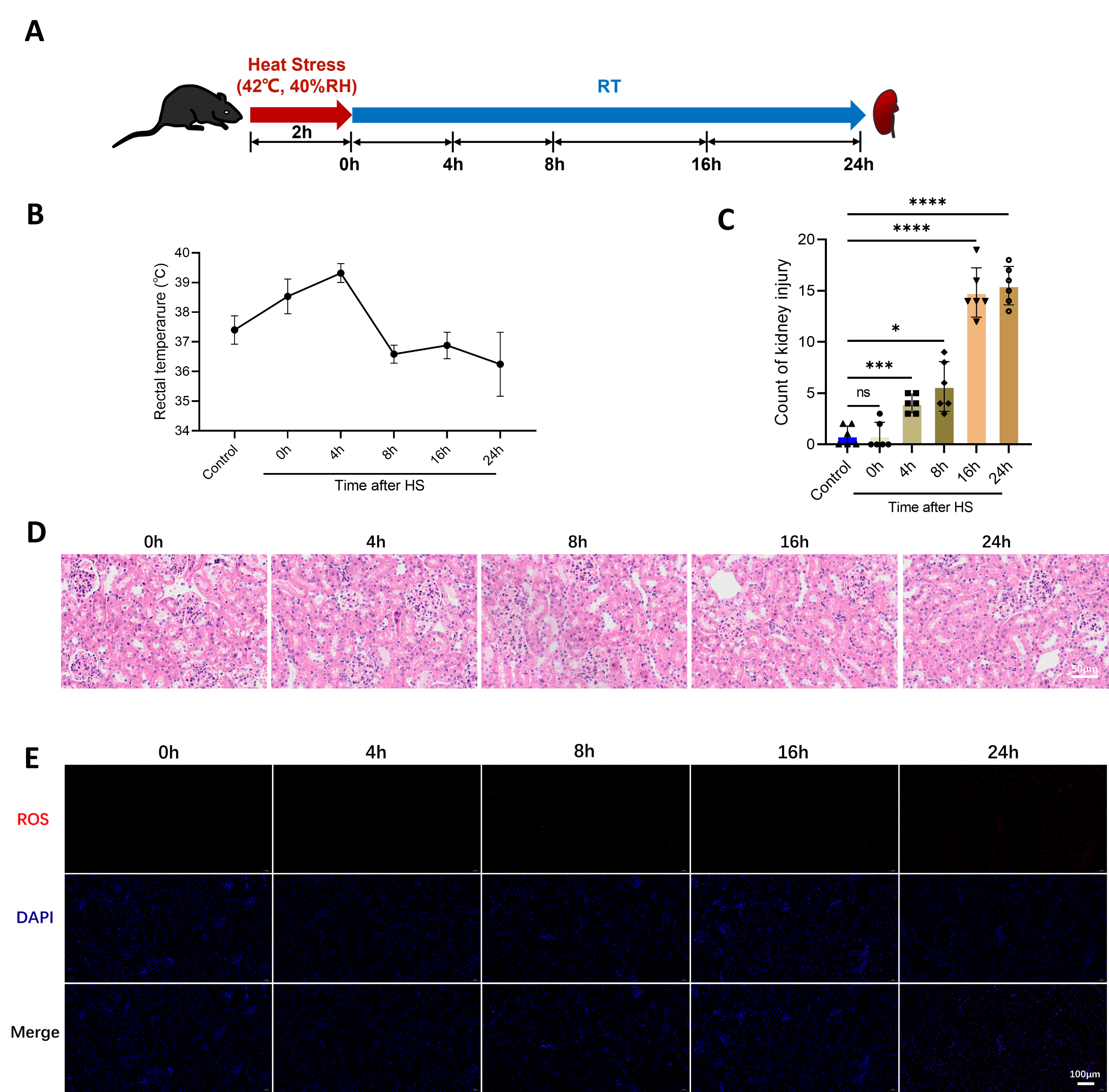


**Figure S1. Acute heat stress induces kidney injury and increases ROS.** *A*, Mice were maintained at 42℃ and humidity at 40 % ± 5 for 2 hours to establish heat stress models. *B*, Body temperature (Rectal temperature) measured before and after acute heat stress treatment at 0h, 4h, 8h, 16h and 24h (there were six mice in each group). *C*, Count of kidney injury before and after acute heat stress treatment at 0h, 4h, 8h, 16h and 24h (there were six mice in each group). *D*, Mice kidneys of Control group were stained with hematoxylin-eosin at different time points after HS (there were six mice in each group). *E*, ROS fluorescence intensity of Control group at different time points after HS (there were six mice in each group).


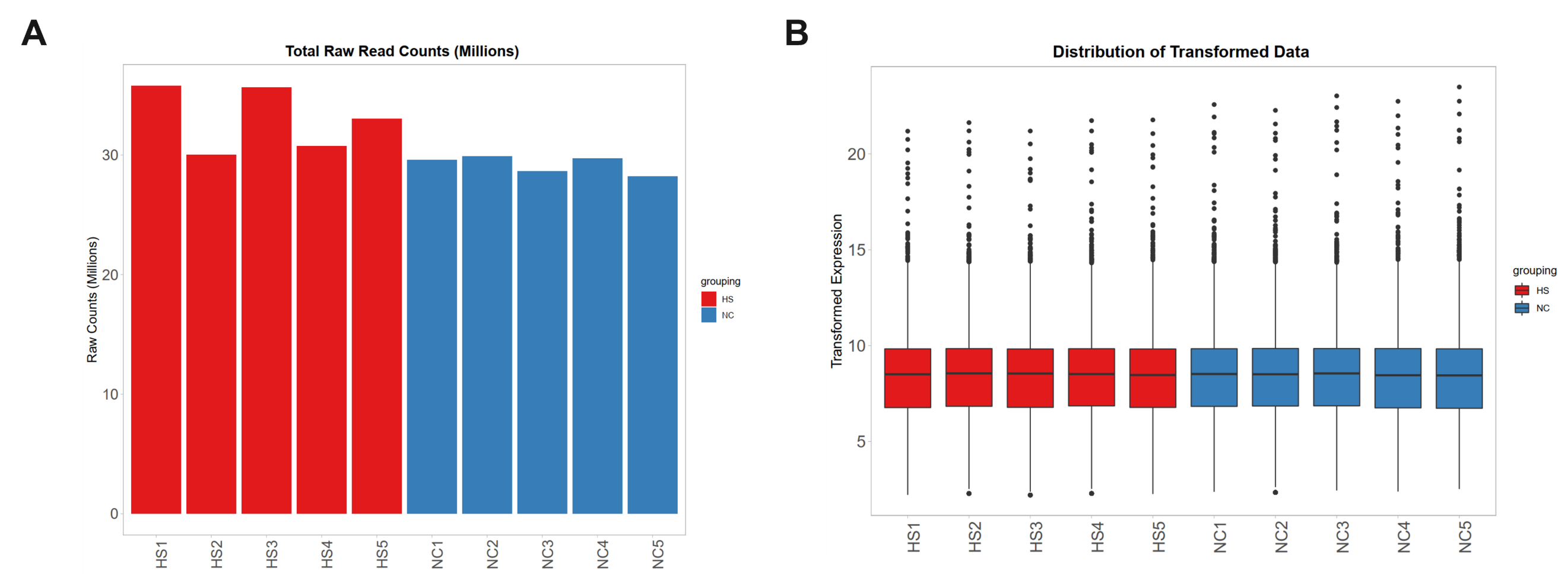


**Figure S2. Data quality analysis.** *A*, Quality control analysis of raw data. *B*, The original data box plot was used to visually detect the sample data dispersion.


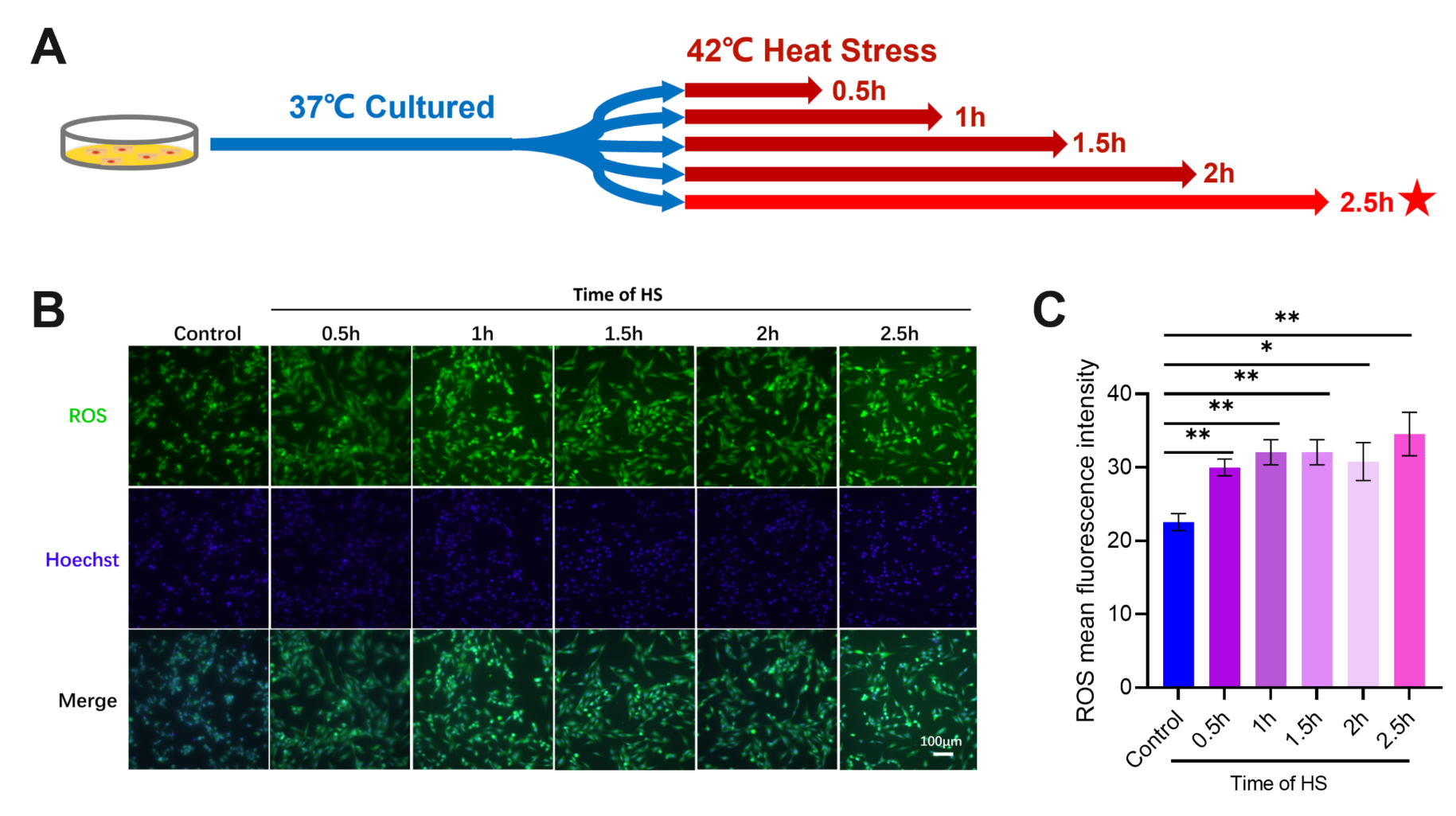


**Figure S3. Construction of TCMK-1 cells model for acute heat stress.** *A*, Flow chart of TCMK-1 cells acute heat stress model construction. *B-C*, ROS fluorescence intensity at different heat stress time of TCMK-1 cells. (Error bars indicate SD, * *p*< 0.05, ***p*<0.01, mean fluorescence intensity related to the amounts of cells)


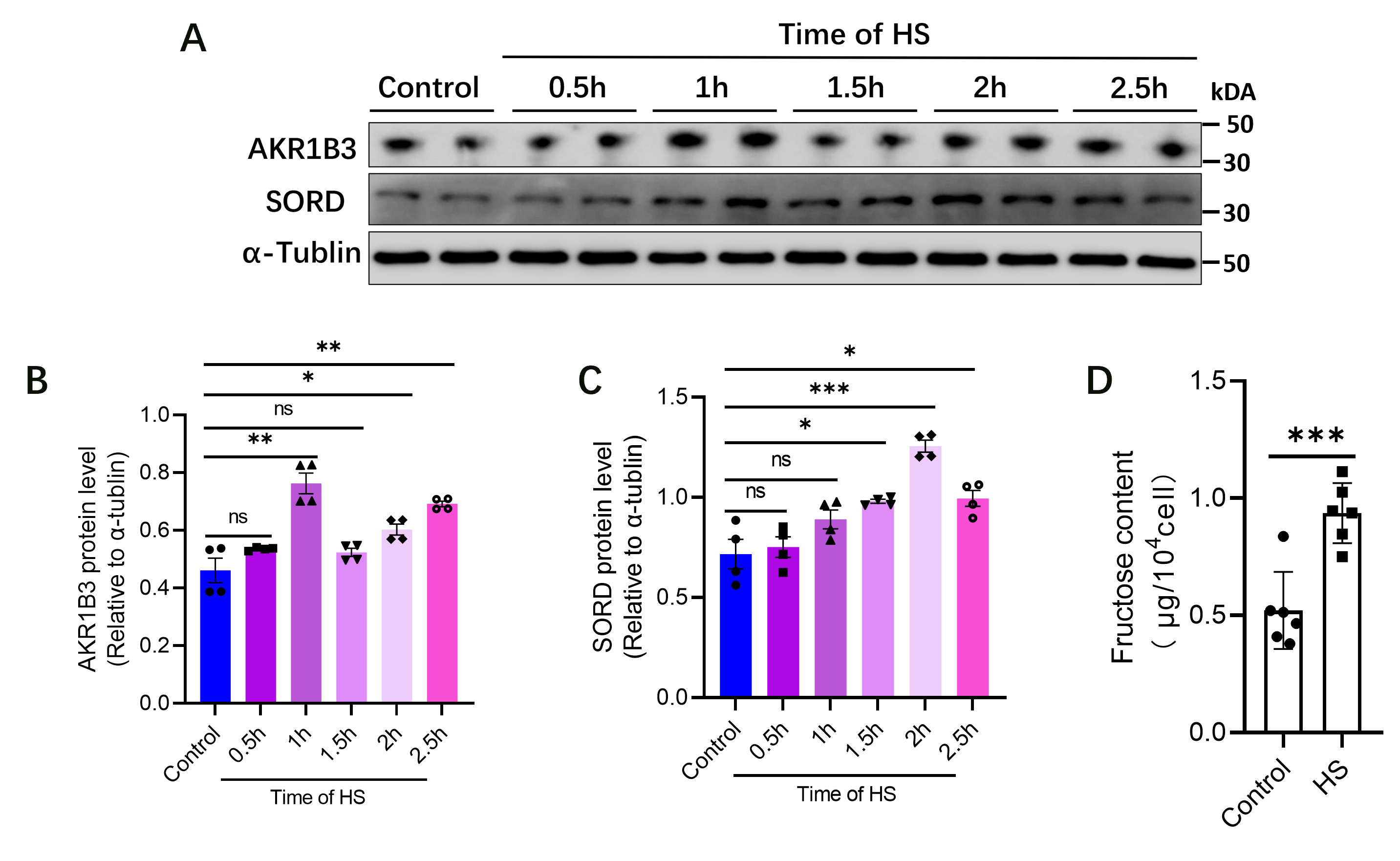


**Figure S4. Changes of the polyol pathway-related enzyme expression and fructose content in TCMK-1 cells after acute heat stress.** *A-E*, The protein expression levels of AKR1B3 and SORD were analyzed by western blotting (Error bars indicate SD, **p*<0.05, ***p*<0.01, ****p*<0.001). *D*, Fructose content in TCMK-1 cells between HS group (heat stress 2.5h) and control group (Error bars indicate SD, ****p*<0.001, there were six mice in each group).


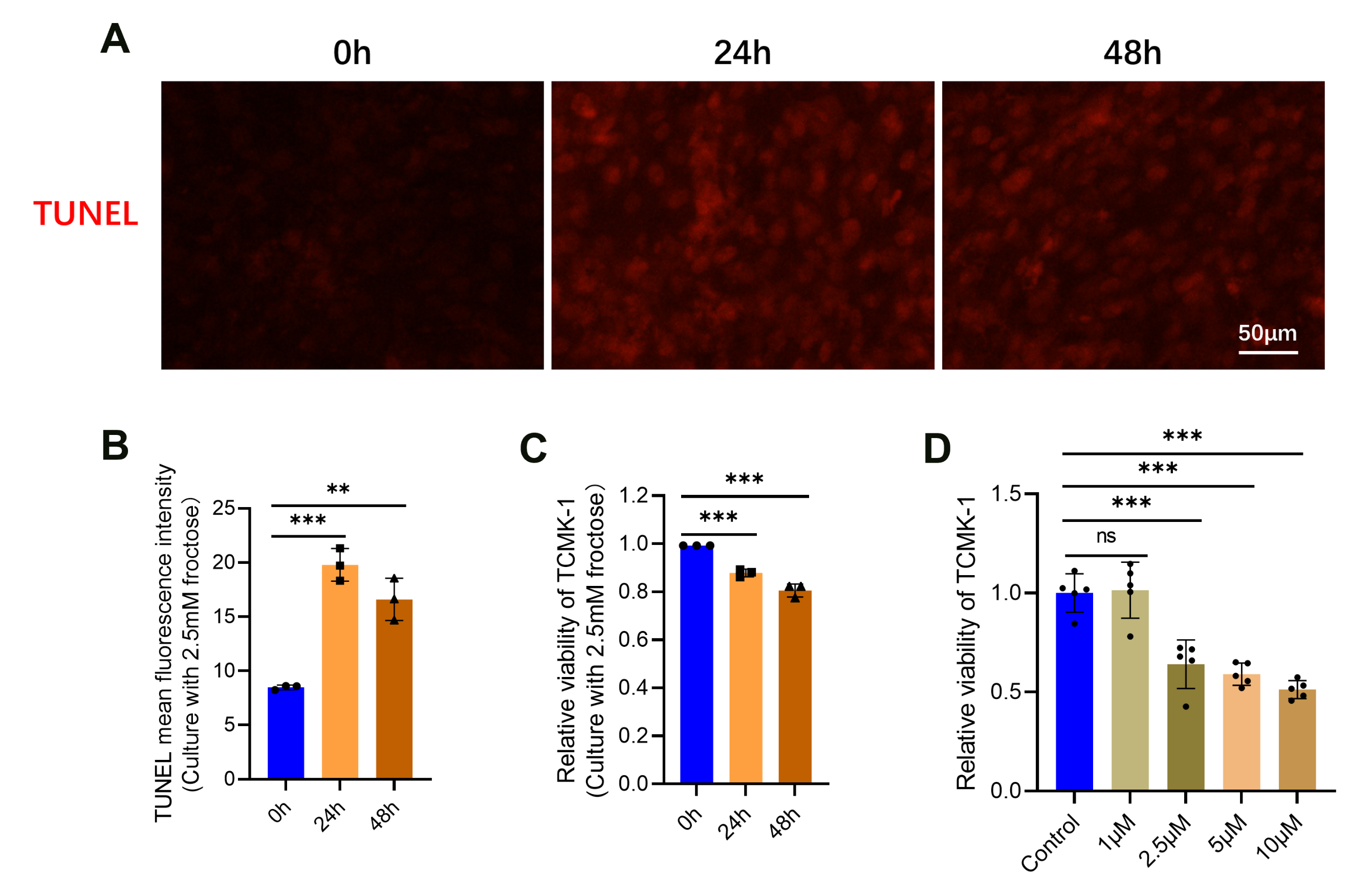


**Figure S5. Increased fructose content decreases cell viability and increases apoptosis.** *A-B*, Fructose treatment increased apoptosis of TCMK-1 cells (Error bars indicate SD, ***p*<0.01, ****p*<0.001). *C*, Fructose treatment decreased TCMK-1 cell viability (Error bars indicate SD, ****p*<0.001).


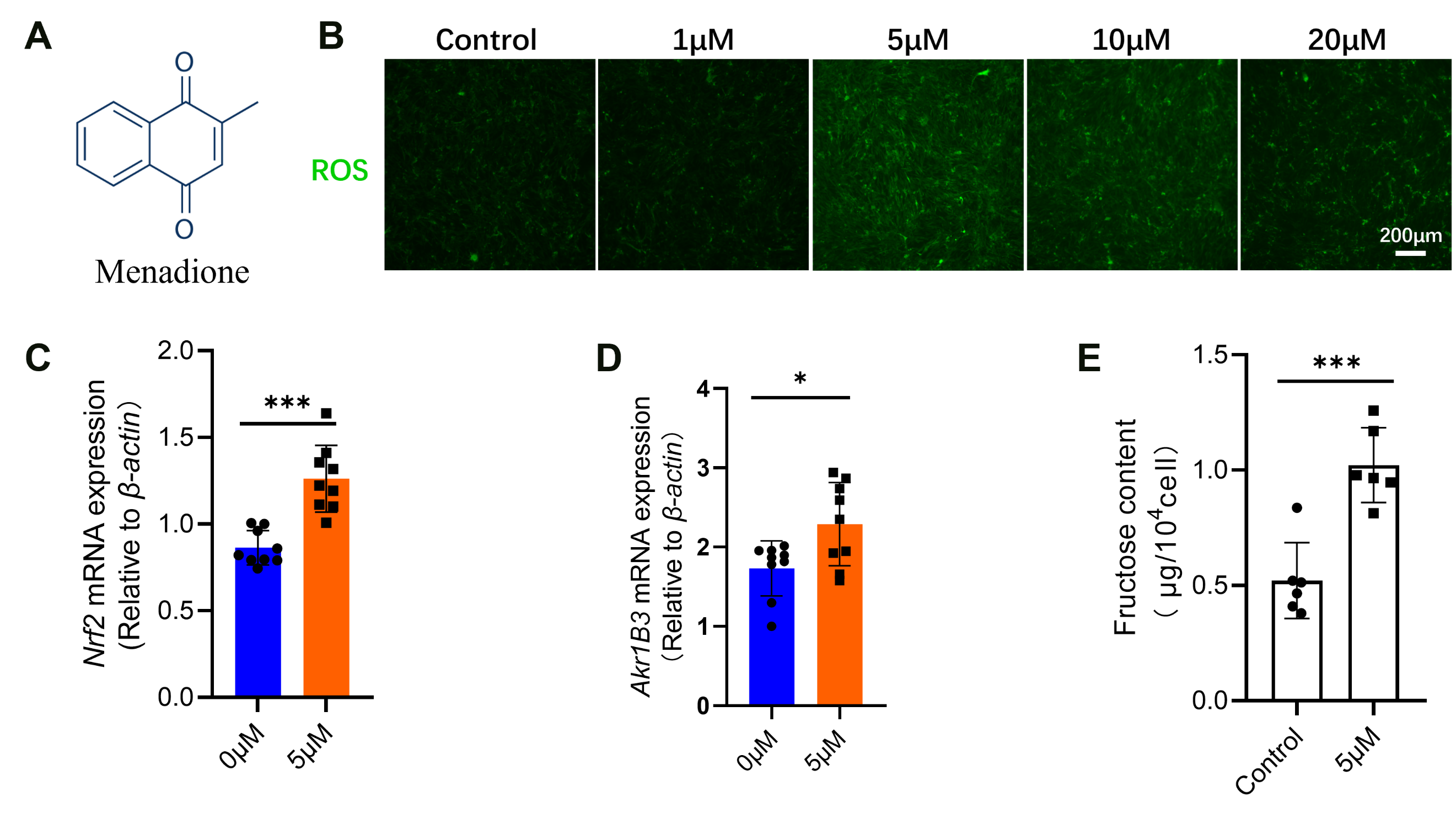


**Figure S6. ROS up-regulates *Nrf-2* and *Akr1b3* and increases intracellular fructose production.** *A*, Molecular structure of Menadione. *B*, Fluorescence intensity of ROS in TCMK-1 treat by different concentration of Menadione. *C-D*, *Nrf-2* and *Akr1b3* mRNA expression change between 0μM group and 5μM group (Error bars indicate SD, **p*<0.05, ****p*<0.001). *E*, Changes of fructose content in TCMK-1 cells between Control group and 5μM group (Error bars indicate SD, ****p*<0.001).


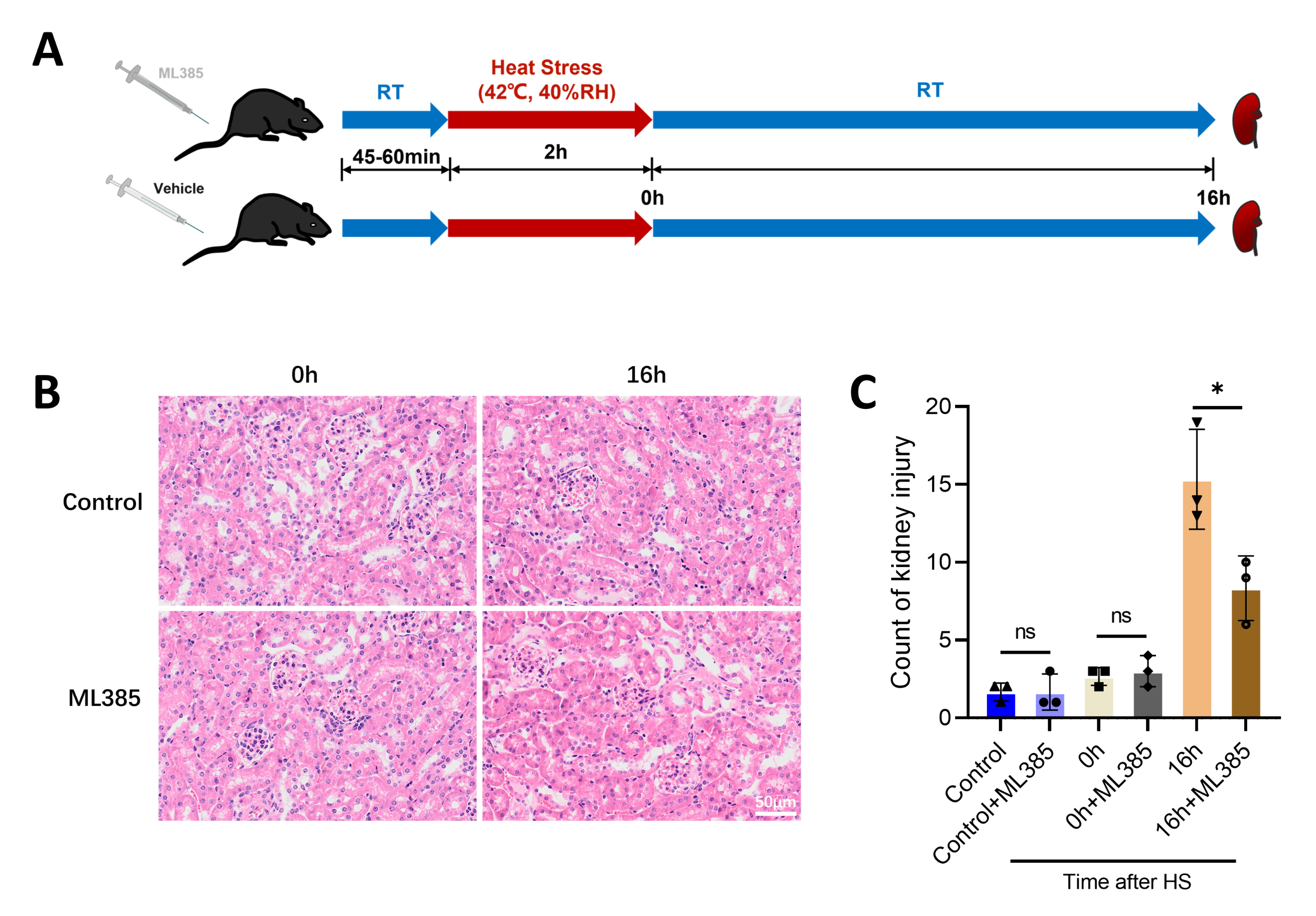


**Figure S7. Inhibition of Nrf-2 restores endogenous fructose production induced by acute heat stress and cell viability after fructose treatment.** *A*, Flow chart of ML385 experimental injection in mice. *B*, Control group at different time points and different treatment (there were three mice in each group). *C*, Count of kidney injury in different treatment (there were three mice in each group).
