# Supplementary figures and images for: Acute heat stress upregulates Akr1b3 through Nrf-2 to increase endogenous fructose leading to kidney injury
Source: J Biol Chem. 2024 Dec 21;301(2):108121. doi: 10.1016/j.jbc.2024.108121 (PMC11834071; doi:10.1016/j.jbc.2024.108121)

**The original blots**


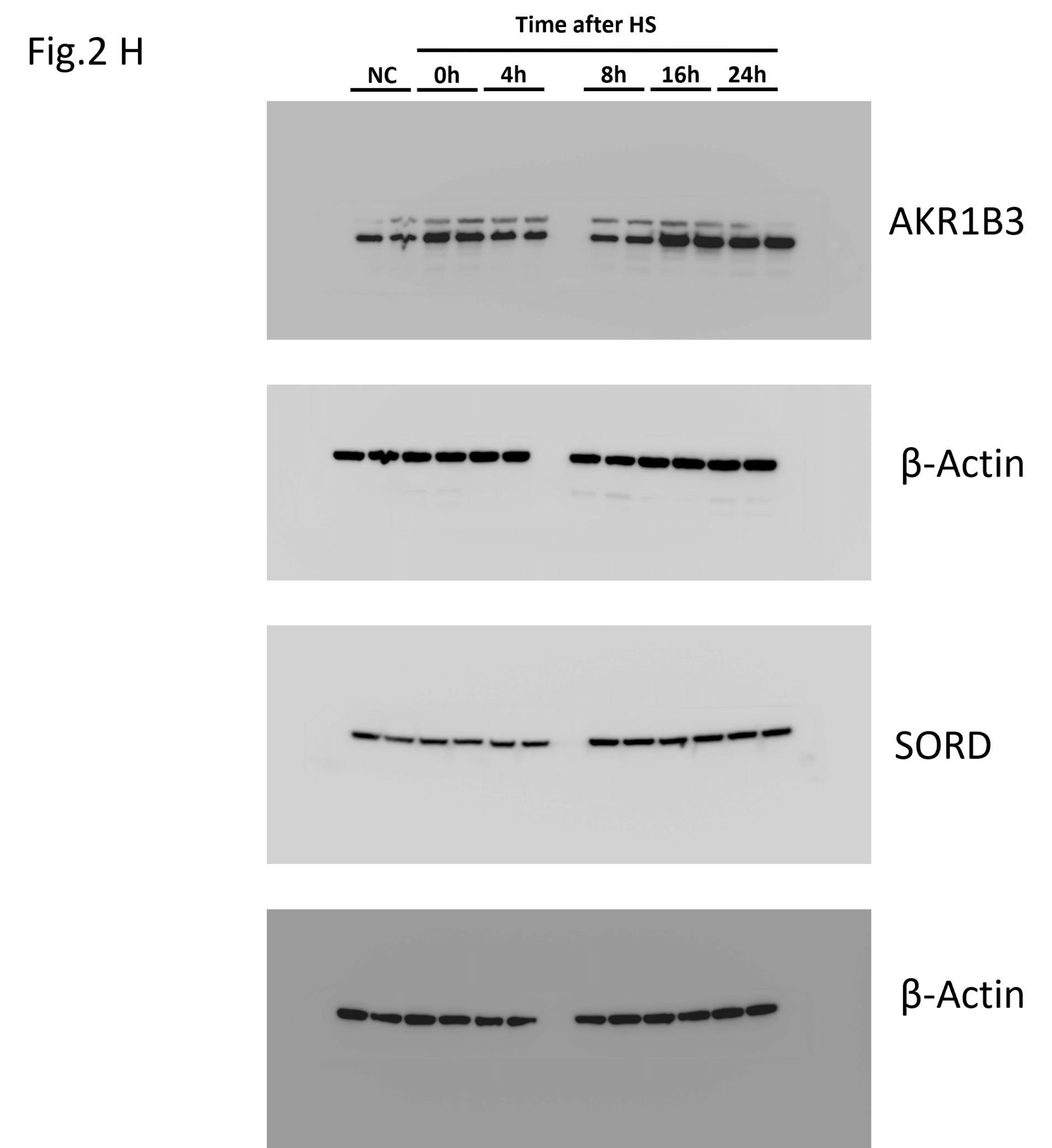

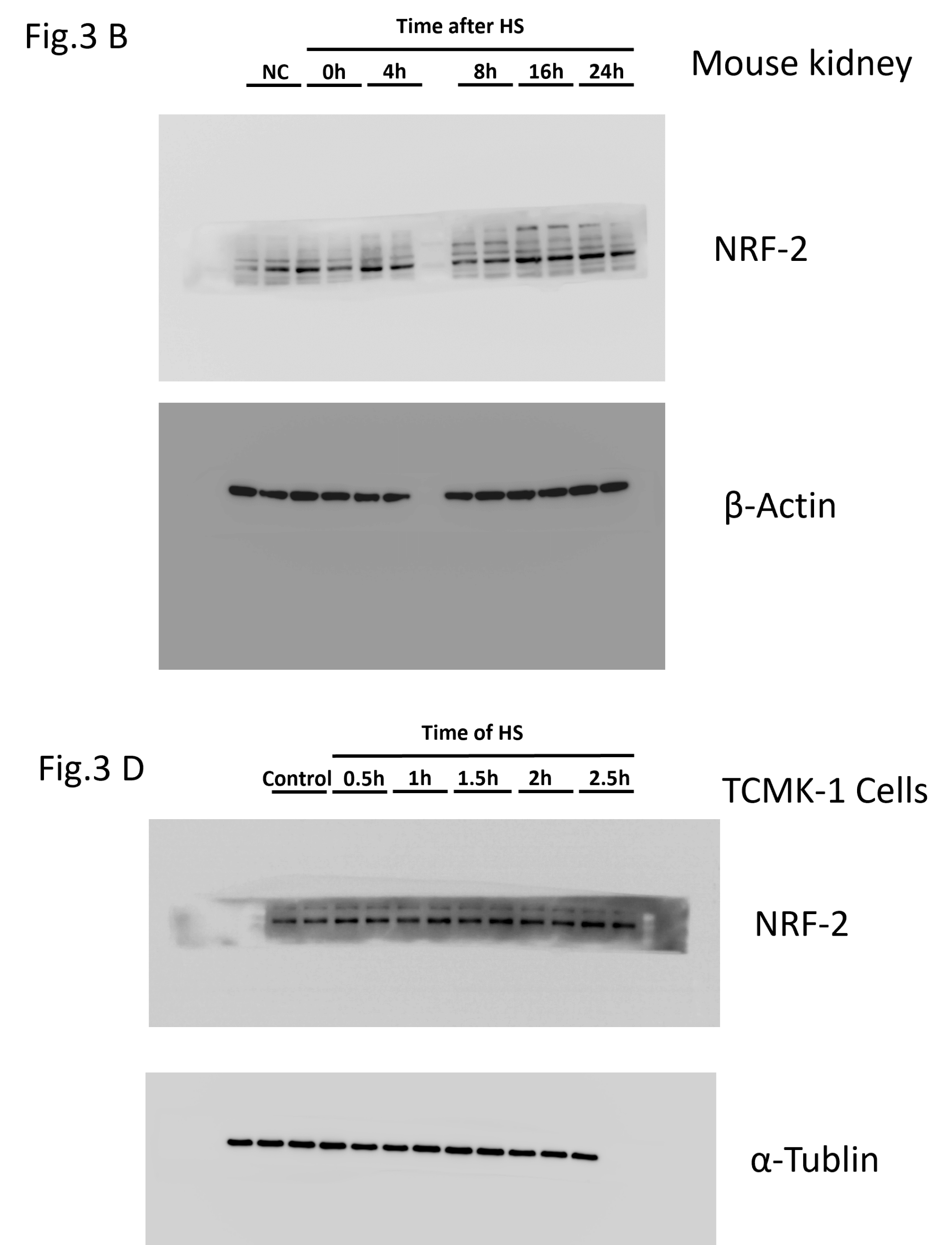

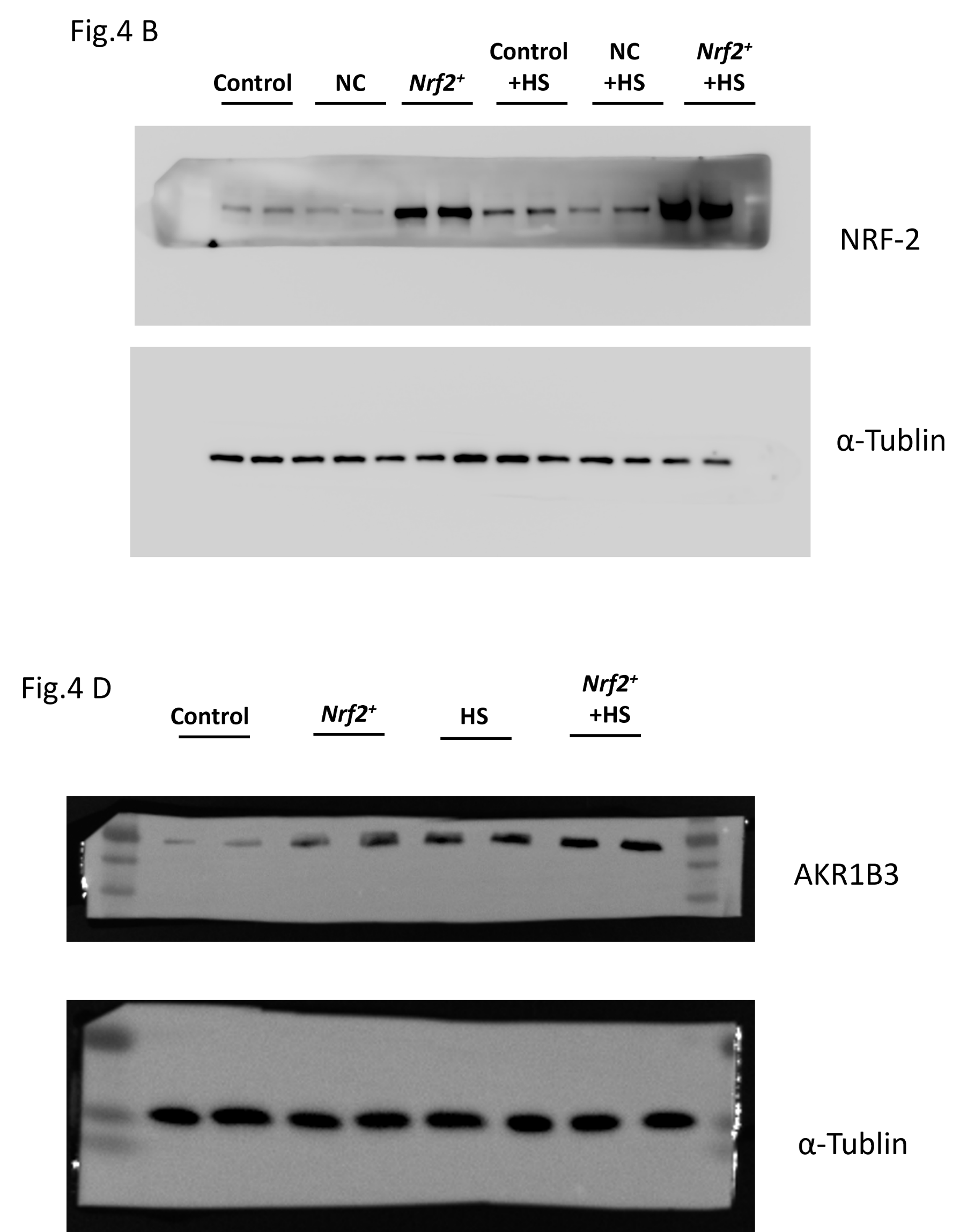

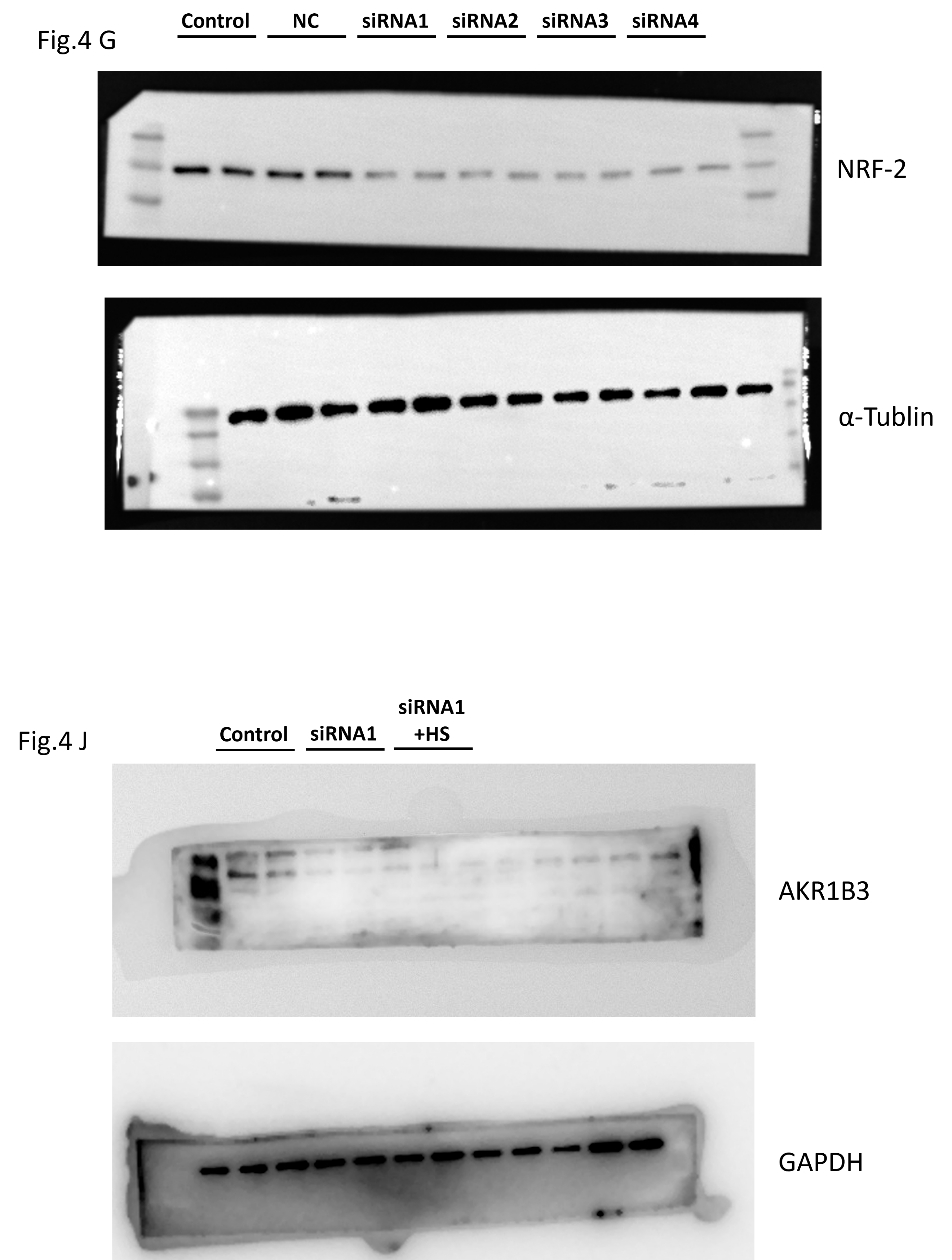

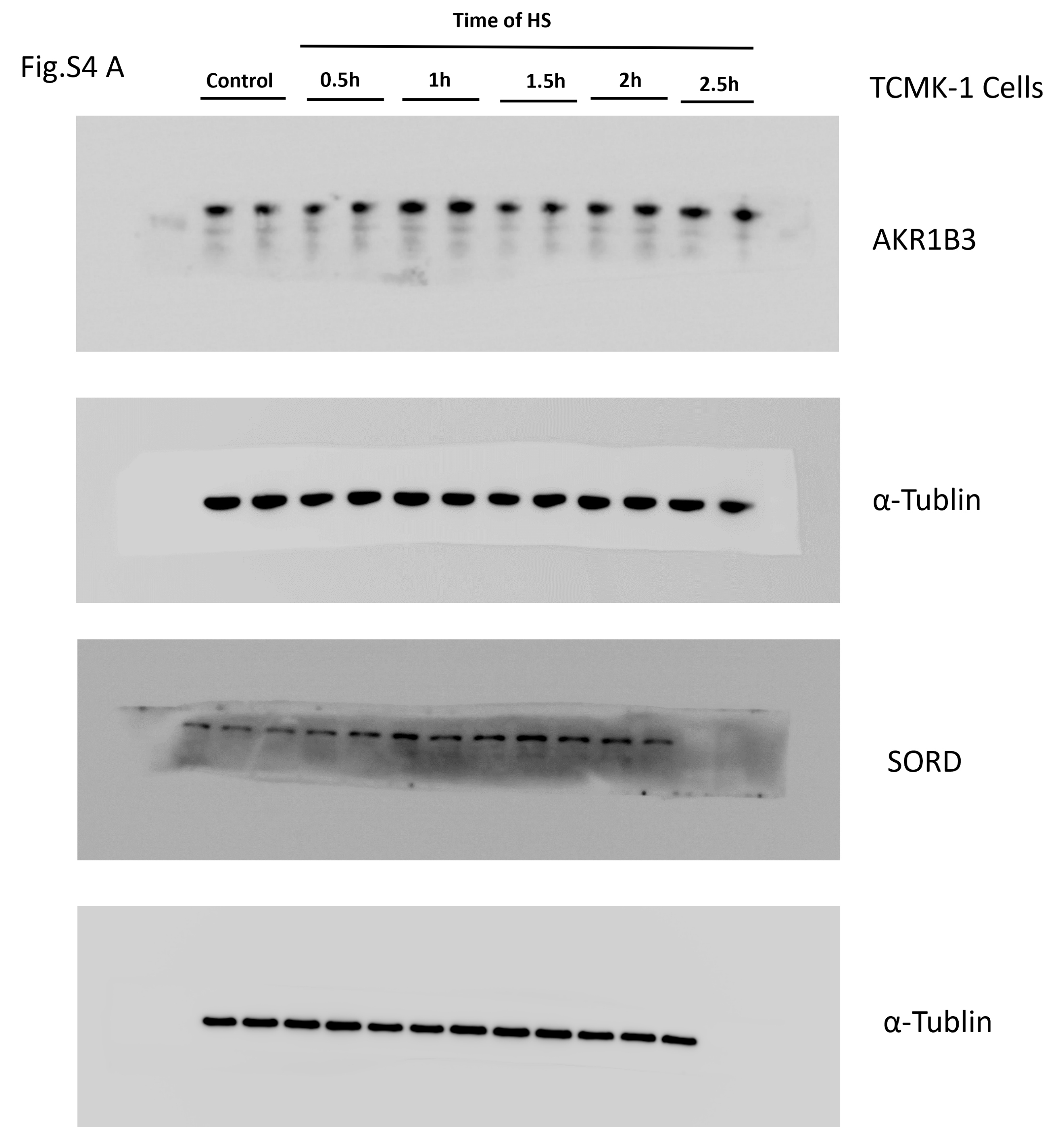

Supplement: Original blots [file mmc3.docx]
